# Meta-analysis links dietary branched-chain amino acids to metabolic health in rodents

**DOI:** 10.1186/s12915-021-01201-2

**Published:** 2022-01-14

**Authors:** Samantha M. Solon-Biet, Lucy Griffiths, Sophie Fosh, David G. Le Couteur, Stephen J. Simpson, Alistair M. Senior

**Affiliations:** 1 Charles Perkins Centre, The University of Sydney, Sydney, NSW Australia; 2 School of Life and Environmental Sciences, Faculty of Science, The University of Sydney, Sydney, NSW Australia; 3 Sydney Medical School, Faculty of Health and Medicine, The University of Sydney, Sydney, NSW Australia; 4 Ageing and Alzheimers Institute and Centre for Education and Research on Ageing, Concord Hospital, Sydney, NSW Australia; 5 ANZAC Research Institute, The University of Sydney, Sydney, NSW Australia; 6 School of Mathematics and Statistics, Faculty of Science, The University of Sydney, Sydney, NSW Australia

**Keywords:** BCAA, Metabolic health, Glucose, Isoleucine, Insulin, Leucine, Meta-regression, Mouse, Obesity, Rat, Valine, Geometric Framework, Nutrition

## Abstract

**Background:**

The role of dietary branched chain amino acids (BCAAs) and their effect on metabolic health is complex. How dietary BCAA levels and their interaction with background nutrition affect health is unclear. Here, we used meta-analysis and meta-regression, together with the nutritional modelling, to analyse the results of rodent studies that increased the level of dietary BCAAs and measured circulating levels, outcomes related to metabolic health, body mass and food intake.

**Results:**

Across all studies, increasing dietary BCAAs resulted in increased levels of circulating BCAAs. These effects, however, were heavily moderated by background dietary levels whereby on high BCAA diets, further increases were not reflected in the blood. Impaired glucose tolerance was associated with elevated dietary BCAAs, with the greatest effect occurring with a simultaneous increase in total protein intake. Effects of dietary BCAAs on plasma glucose, insulin, or HOMA emerged only when dietary macronutrient background was considered. We found that elevated dietary BCAAs increases % body fat, with largest increases in adiposity occurring when BCAAs are increased on a high protein, low carbohydrate dietary background. Finally, we found that increased dietary BCAAs were associated with increased food intake when the background diet was low in BCAAs.

**Conclusion:**

Our data highlights the interaction between BCAAs and background nutrition. We show that the effects of BCAAs on metabolic health cannot be studied in isolation but must be considered as part of complex mixture of dietary components.

**Supplementary Information:**

The online version contains supplementary material available at 10.1186/s12915-021-01201-2.

## Background

The relationship between dietary branched chain amino acids (BCAAs), blood levels of BCAAs and their effects on body composition and metabolic health is gaining increasing attention. The interaction between BCAAs and health, however, is complex and the literature inconsistent. Studies in humans and animals have yielded conflicting outcomes and conclude that dietary BCAAs and/or blood levels of BCAAs have either positive or negative impacts on body composition and metabolic health [1–10]. In humans, many studies have shown an association between increased circulating levels of BCAAs and obesity, insulin resistance and type 2 diabetes [9, 11, 12]. Additionally, circulating BCAAs have also been postulated as a predictive biomarker of future type 2 diabetes [6, 9, 13]. In contrast, other studies have shown that BCAA supplementation, particularly in the context of undernutrition and ageing, has positive effects on health and lifespan [3, 14, 15].

This complexity, in part, reflects the physiology and regulation of BCAAs, which are both signalling molecules and nutrients. As essential amino acids, the BCAAs, isoleucine, leucine and valine are acquired primarily through the diet. Unlike other amino acids, there is no hepatic metabolism of dietary BCAAs; therefore in the postprandial phase, blood levels of BCAAs directly correspond with dietary intake of BCAAs [16]. However, beyond the postprandial period and during fasting, blood levels of BCAAs are tightly regulated through catabolism by branched chain α-ketoacid dehydrogenase complex (BCKDH) [17, 18]. Insulin also plays a key role in the regulation of BCAAs and their impact on metabolism. Together, insulin and BCAAs influence protein synthesis through activation of MTOR, BCAA catabolism through activation of BCKDH and the control of food intake [17]. Any relationship between dietary BCAAs and blood levels of BCAAs can therefore be influenced by many factors, including the timing of the blood samples, the BCAA content of the food, endogenous factors such as insulin which regulate BCAA metabolism and degradation, and numerous comorbid conditions (such as obesity, diabetes, renal failure, liver cirrhosis, cancer, sepsis) that influence BCAAs independently of dietary BCAA content [19].

This network of pathways linking BCAAs, insulin and metabolism has provided plausible mechanisms for some of the paradoxical findings in humans. For example, the association between obesity and elevated blood levels of BCAAs has been explained by the finding that insulin resistance causes impaired degradation of BCAAs [9]. On the other hand, body lean mass has also been found to be positively associated with blood levels of BCAAs, with the proposed mechanism being the activation of MTOR by BCAAs leading to increased muscle protein [1, 17].

Further confounding these associations is the complexity of diet. Diet is a mixture of many nutrients that vary enormously between individuals and cultures [20]. A diet that is high in BCAAs from plant sources may have different effects on health than a diet that is high in BCAAs from animal sources, yet both diets may be equal in terms of BCAA content. The underlying diet can also influence the physiological impact of BCAAs. A diet that is high in BCAAs and high in all other essential amino acids will lead to increased protein synthesis. Yet a diet that is high in BCAAs and low in the other essential amino acids will not lead to protein synthesis despite MTOR activation, because protein synthesis requires all amino acids [21]. In addition to the dietary BCAA content, any effects of dietary BCAAs on health and metabolism may be influenced by many of the other components of the underlying diet. For example, the background level of carbohydrate, relative to dietary BCAA or protein content, can moderate any effects on metabolic health. In mice, for example, diets that are low in carbohydrate but high in BCAAs accelerated markers of ageing, such as MTOR and IGF1, whereas elevating BCAAs against a high-carbohydrate background did not [10, 22].

Resolving these paradoxes and complexities requires answers to two pivotal questions: (1) Do circulating BCAAs reflect dietary intakes? (2) Are there metabolic or body composition effects of dietary BCAAs, and if so, are these moderated by nutrient background? Determining the answers to these questions is difficult in human studies because these are confounded by a lack of precision in dietary intake data and various factors including differences in diets, socioeconomic factors and underlying diseases/obesity that can also influence blood BCAA levels of independent of diet. Here, we use animal data to overcome the methodological limitations in observational human studies. For the first time, we bring together established methods in meta-analysis and meta-regression with the powerful geometric framework for nutrition (GFN) [20] to model the complex relationship between diet, BCAAs and metabolic health. We show that in both the fasting and fed state, circulating BCAA levels reflect levels of BCAAs in diet. There is, however, a saturating effect, where circulating BCAA levels plateau as dietary levels increase beyond a point, reflecting their systemic physiological effect. We also show that the effect of dietary BCAAs on health is complex and is dependent on the dietary background upon which BCAAs are manipulated.

## Results

### Circulating BCAAs reflects dietary levels

**Fig. 1.**
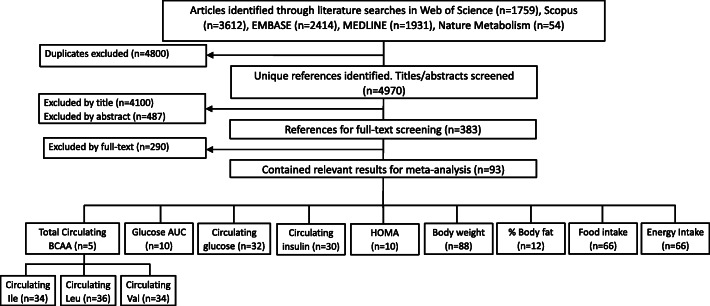
PRISMA-style flow diagram illustrating the inclusion and exclusion of studies from our literature search. The number of studies (*n*) at each stage is given. For reference, a full list of included references in each analysis is given in Additional File 1: Table S1 and Table S6.

To determine the relationship between dietary BCAAs and circulating levels, we extracted data on circulating levels of total BCAAs (Fig. 1; 52 diet groups; 5 articles), isoleucine (165 diet groups; 34 articles), leucine (168 diet groups; 36 articles) and valine (155 diet groups; 34 articles) (Additional File 1: Table S1). For total BCAA levels, 64% of the data came from groups of mice, for isoleucine and leucine 50% of the groups were mice, and valine 57% were mice.

**Fig. 2.**
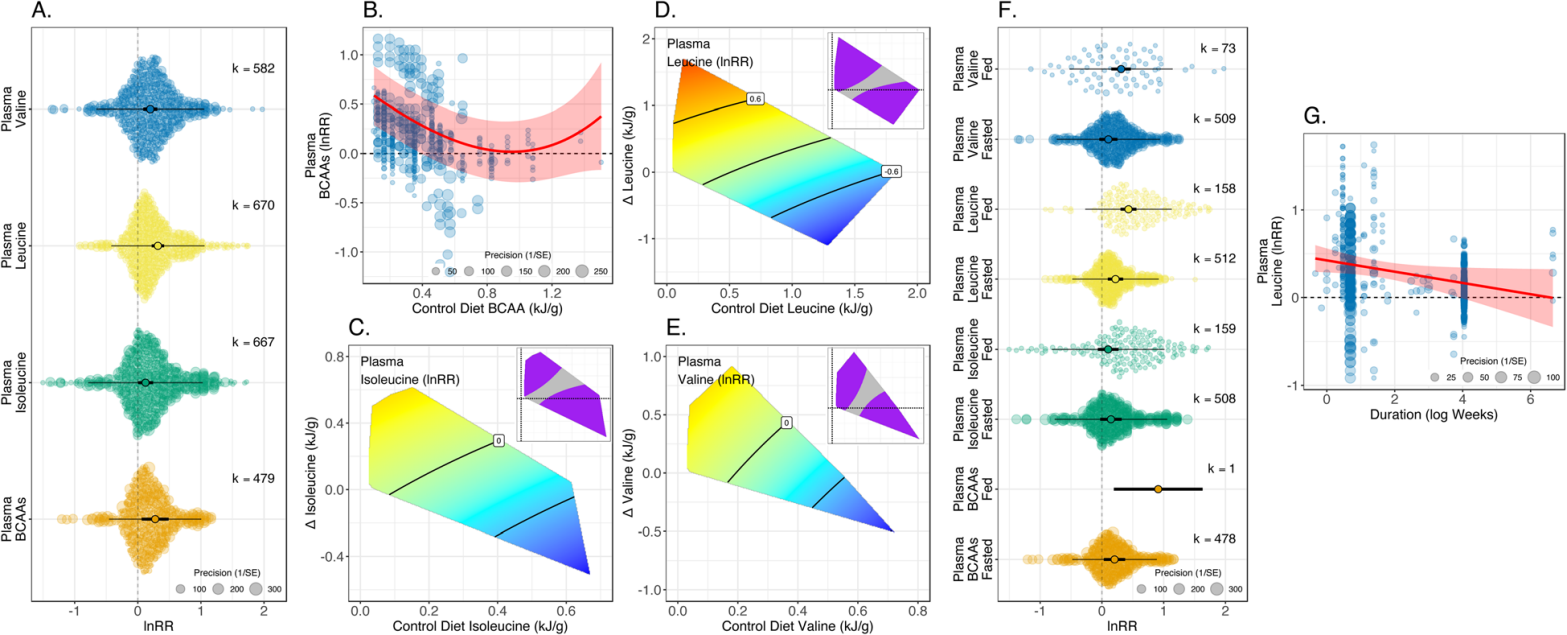
**A** Orchard plots showing mean effects of increased dietary BCAAs on plasma levels of BCAAs (total and individual). Thick error bars are 95% confidence intervals (CI; i.e. statistical significance) and fine error bars are 95% prediction intervals (i.e. heterogeneity in reported effects; the range within which 95% of effect sizes are expected to fall), and *k* is the number of effect sizes (lnRR). Positive effects indicate that the outcome measure is higher on the experimental diet (with higher BCAAs) than the control diet. **B** lnRR for total plasma BCAAs as a function of the BCAA content of the control diet. Red line indicates the fitted values from meta-regression, with the shaded area the CI. **C**–**E** Surfaces showing meta-regression estimates of lnRR for plasma levels of individual BCAAs as a function of the difference in dietary levels between experiment and control, and overall control levels of the focal amino acid. On surfaces, red colours indicate positive effects, blue colours negative effects and purple areas on inlaid panels indicate that the CI for that region of the surface does not span zero. All fitted values come from the AIC favoured-model (see Additional File 1: Table S5 for coefficients). **F** Orchard plots showing effects for each outcome stratified by whether animals were fasted or fed prior to sampling (the significance of between group contrasts are given in Additional File 1: Table S3). **G** Where shown, individual effect sizes are scaled by their precision

Meta-analysis applied to all pairwise comparisons among diets within a controlled experiment detected statistically significant positive effect sizes for all measures of circulating BCAAs (Fig. 2A). Back transforming the overall effects from meta-analysis suggests that increasing dietary BCAAs results in plasma levels of 132% total BCAAs, 113% isoleucine, 137% leucine and 122% valine that in control rodents (Fig. 2A). However, for all outcomes, high levels of heterogeneity were detected (*I*^2^_Total_ > 99%; Table 1) suggesting substantial variation in the reported effect sizes. Only small to moderate amounts of this variation were attributable to among-study differences (*I*^2^_Experiment_ = 26 to 36%; Table 1).

**Table 1:** Overall effects (lnRR), 95% confidence intervals (CI) and heterogeneity statistics as estimated by multi-level meta-analysis. *HOMA* Homeostatic Model Assessment (a measure of insulin sensitivity)

Trait	lnRR	CI	*σ*^2^_Experiment_	*σ*^2^_Residual_	*I*^2^_Total_	*I*^2^_Experiment_
Plasma BCAAs	0.275	0.061 to 0.489	0.035	0.103	99.87	25.54
Plasma isoleucine	0.122	0.003 to 0.241	0.077	0.137	99.91	35.78
Plasma leucine	0.319	0.221 to 0.416	0.046	0.094	99.14	32.69
Plasma valine	0.198	0.086 to 0.310	0.054	0.136	99.81	28.41
Glucose AUC	0.193	0.072 to 0.314	0.010	0.093	84.21	8.53
Plasma glucose	0.004	− 0.036 to 0.043	0.007	0.006	79.26	41.51
Plasma insulin	0.038	− 0.056 to 0.131	0.004	0.183	83.02	1.71
HOMA	0.019	− 0.108 to 0.146	0.000	0.243	74.81	0.00
Mass	0.010	− 0.024 to 0.043	0.022	0.024	98.43	47.49
Percent fat mass	0.017	− 0.106 to 0.140	0.014	0.063	89.63	16.07
Food intake	− 0.067	− 0.106 to − 0.028	0.018	0.027	94.45	37.67
Energy intake	− 0.058	− 0.093 to − 0.023	0.013	0.044	99.98	22.37

Meta-regressions of nutritional moderators suggested that the effect sizes for any one measure of circulating levels of BCAAs could be predicted by the dietary context (see Additional File 1: Table S2 for relative AIC of different dietary models). For plasma levels of total BCAAs, elevating dietary BCAAs on a diet low in BCAAs resulted in big increases in circulating levels (Fig. 2B). However, where diets are already high in BCAAs, further increases are not predicted to result in much, if any, change (Fig. 2B). For circulating levels of the individual BCAAs themselves, response surface showed there was an interaction between the magnitude of the dietary increase and the levels already present in the diet. Large increases of a specific BCAA where the diet is low in that amino acid result in big increases in circulating levels, and *vice versa* (Fig. 2C–E).

Most of the data came from studies that fasted animals prior to serum sampling. Overall, the effects of dietary BCAAs on circulating levels were larger in fed than fasted animals (Fig. 2F). However, fasting status was only a statistically significant moderator for Leucine (lnRR_Fed-Fasted_=0.209, CL=0.035 to 0.383; see Additional File 1: Table S3 for between-group comparisons of non-nutritional moderator variables). There was no statistically significant difference between effect sizes in mice and those in rats. For all the different circulating levels of BCAAs, the duration of exposure was estimated to have a negative effect, although this was only significant for circulating leucine (Fig. 2G; Additional File 1: Table S3).

Egger’s regression indicated publication bias may be present for circulating levels of total BCAAs, leucine and valine (see Additional File 1: Table S4 for output of publication bias tests). For total BCAAs, leucine and valine, trim and fill analyses indicated 120, 8 and 133 missing effect sizes, respectively. For total BCAAs and valine, inclusion of any predicted missing effects was estimated to increase overall effects (total BCAAs lnRR_Adjusted_ = 0.318 or 137%, valine lnRR_Adjusted_ = 0.349 or 142%). For leucine the inclusion of missing studies was estimated to slightly decrease the overall effect (lnRR_Adjusted_ = 0.235 or 126%).

Taken together, these results suggest that, while there may be some differences among studies, circulating BCAAs are increased by adding dietary BCAAs when animals are restricted to a base diet that is low to moderate in BCAAs. Above a particular concentration of BCAAs in the diet (approximately 0.5 kJ/g), blood levels do not increase further as BCAA content of the diet increases. Fasting animals can reduce the size of the effect of dietary BCAAs on circulating plasma BCAA levels but is not likely to abrogate it completely. There is also some mixed evidence that over time, the effect of dietary BCAAs on circulating levels may become less extreme. This suggests that circulating levels do not simply reflect diet, but that dietary BCAAs have a systemic physiological effect on amino-acid metabolism.

### Dietary BCAAs, glucose homeostasis and insulin sensitivity

To evaluate how dietary and circulating BCAAs influenced glucose homeostasis, we gathered data on the area under the curve (AUC) in a glucose tolerance test (Glucose AUC; 57 diets from 10 articles), plasma levels of glucose (100 diets from 32 articles) and insulin (115 diets from 30 articles) and HOMA (55 diets from 10 articles; Fig. 1). For estimates of AUC, 98% of glucose data came from mice. For plasma levels, 51% of glucose estimates came from groups of mice, and insulin 89% were from mice. Mice contributed 95% of the estimates of HOMA.

**Fig. 3.**
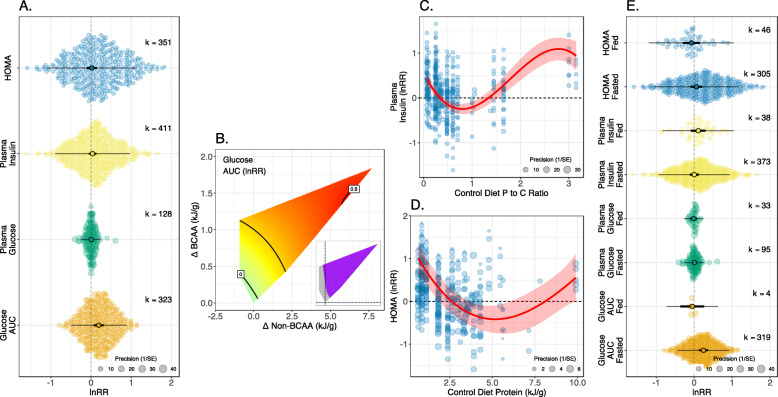
**A** Orchard plots showing mean effects of increased dietary BCAAs on indicators of glucose metabolism. Thick error bars are 95% confidence intervals (CI; i.e. statistical significance) and fine error bars 95% prediction intervals (i.e. heterogeneity in reported effects; the range within which 95% of effect sizes are expected to fall), and *k* is the number of effect sizes (lnRR). Positive effects indicate that the outcome measure is higher on the experimental diet (with higher BCAAs) than the control diet. **B** Surface showing meta-regression estimate of lnRR for glucose AUC as a function of the difference in dietary BCAA and non-BCAA levels between experimental and control diets. **C**, **D** Bubble plots for lnRR of plasma insulin and HOMA as a function nutritional moderators. Red line indicates the fitted values from meta-regression, with the shaded area the CI. On surfaces, red colours indicate positive effects, blue colours negative effects and purple areas on inlaid panels indicate that the CI for that region of the surface does not span zero. All fitted values come from the AIC-favoured model (see Additional File 1: Table S5 for coefficients). **E** Orchard plots showing effects for each outcome stratified by whether animals were fasted or fed prior to sampling (the significance of between group contrasts are given in Additional File 1: Table S3)

Meta-analysis of all pairwise diets within an experiment detected a significant positive effect for glucose AUC, but not any other traits related to glucose homeostasis (Table 1, Fig. 3A). Glucose AUC of rodents on high BCAA diets was 121% of that of animals on lower BCAA diets. For all traits, there was relatively high heterogeneity (*I*^2^_Total_ = 75–84%; Table 1), with low to moderate amounts attributable to among experiment-level differences (*I*^2^_Experiment_ = 0–42%; Table 1).

For glucose AUC, a model fitting an interaction between differences in dietary levels of BCAAs and non-BCAAs had the best fit based on AIC (Additional File 1: Table S2). Using response surfaces in the GFN to plot predicted effect sizes, we saw that increasing BCAAs and non-BCAAs simultaneously (i.e. increasing protein content), resulted in the largest effects of dietary BCAAs on glucose AUC (Fig. 3B). The effects of dietary BCAAs on plasma insulin levels were best predicted by the protein to carbohydrate ratio of the diet, in a U-shaped manner; at both low and high protein to carbohydrate ratios, increasing dietary BCAAs increased plasma insulin levels (Fig. 3C). Similarly, dietary protein was estimated to have a U-shaped moderating effect of BCAAs on HOMA (Fig. 3D). Dietary BCAAs positively influenced HOMA when diets were both low and high in dietary protein. For plasma glucose levels, no nutritional moderator had lower AIC than the (null) meta-analysis. Neither fasting status, model species nor duration of the study was a significant moderator of effect size for any glucose metabolism outcomes (Fig. 3E, Additional File 1: Table S3).

Egger’s regression indicated publication bias for plasma insulin and HOMA (Additional File 1: Table S4). Trim and fill analysis applied to the effect sizes for these traits estimated 73 and 57 missing studies for plasma insulin and HOMA respectively, and inclusion of such missing studies is estimated to reduce estimated lnRR slightly. It is notable that overall meta-analytic means for plasma insulin and HOMA traits are already non-significant, although the respective model estimates shown in Fig. 3A may require slight downward adjustment due to publication bias (plasma insulin lnRR_Adjusted_ = −0.063 or 94%, HOMA lnRR_Adjusted_ = −0.011 or 99%).

These results suggest that any effects of increasing BCAAs are dependent on the dietary background upon which the change occurs. While slightly different moderators of effect were favoured for the different traits, a recurring theme is dietary protein. Increasing BCAAs on a diet already low/high in protein is most likely to result in poorer glucose and insulin homeostasis.

### BCAAs, food intake and body composition

Given evidence that BCAAs influence food intake and body composition [10], we gathered data on body mass from 435 groups of rodents on different diets (88 articles) and percent fat mass from 58 dietary groups (12 articles). We also had estimates of food and energy intake from 338 groups (66 articles). For body mass, 31% of the groups were mice, for percent fat mass 97% were mice, and for food and energy intake 36%.

**Fig. 4.**
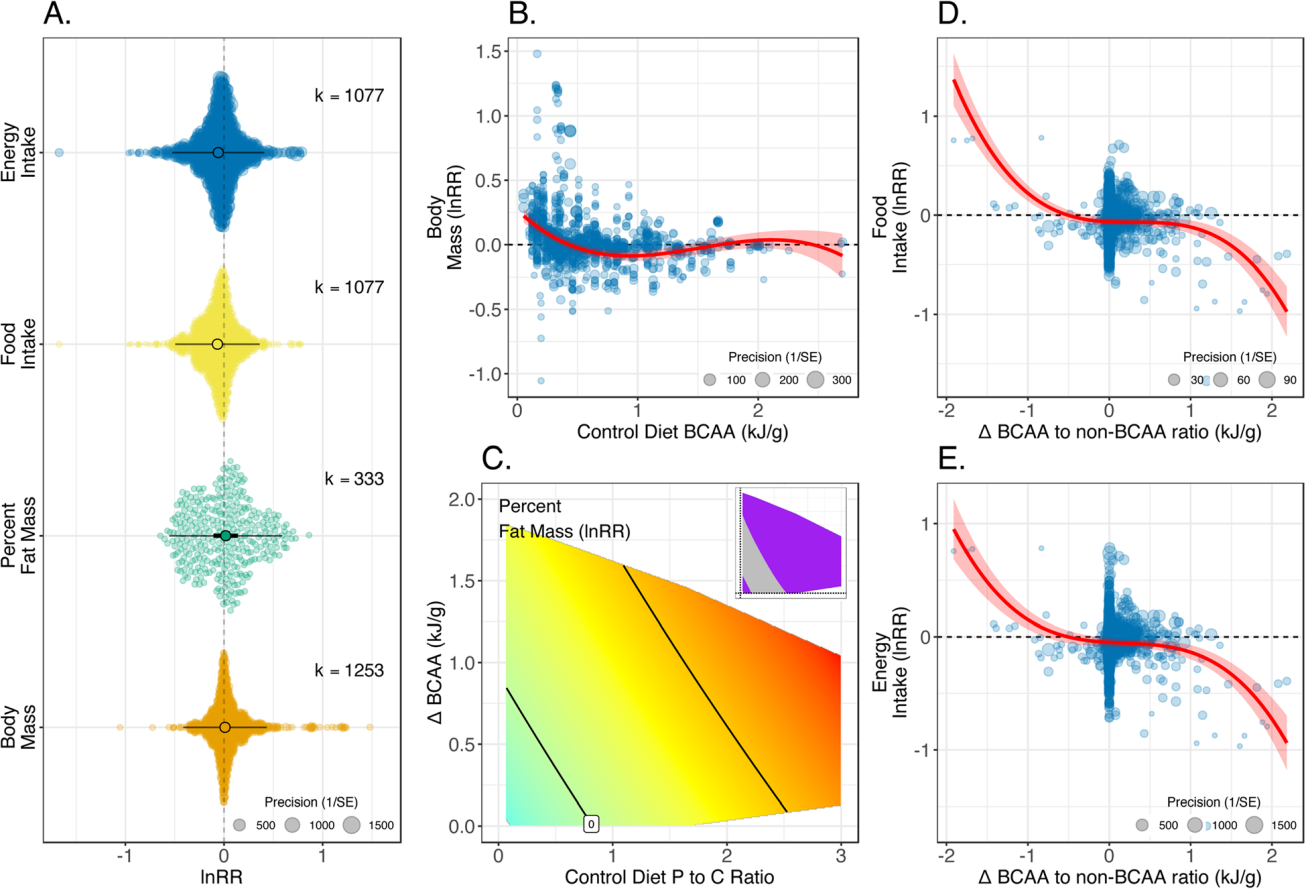
**A** Orchard plots showing mean effects of increased dietary BCAAs on indicators of body composition and food intake. Thick error bars are 95% confidence intervals (CI; i.e. statistical significance) and fine error bars 95% prediction intervals (i.e. heterogeneity in reported effects; the range within which 95% of effect sizes are expected to fall), and *k* is the number of effect sizes (lnRR). Positive effects indicate that the outcome measure is higher on the experimental diet (with higher BCAAs) than the control diet. **B**, **D**, **E** Bubble plots for lnRR of body mass, food and energy intake as a function of nutritional moderators. Red line indicates the fitted values from meta-regression, with the shaded area the CI. **C** Surface showing meta-regression estimate of lnRR for percentage fat mass as a function of the difference in the ratio of dietary BCAA:non-BCAA between experimental and control diets and the protein to carbohydrate ratio of the control diet. On surfaces, red colours indicate positive effects, blue colours negative effects and purple areas on inlaid panels indicate that the CI for that region of the surface does not span zero. All fitted values come from the AIC-favoured model (see Additional File 1: Table S5 for coefficients). Where shown, individual effect sizes are scaled by their precision

There was no overall significant effect size for body mass or percent body fat (Fig. 4A; Table 1). However, overall effect sizes for food and energy intake were negative and statistically significant suggesting that, on average, intake is lower on high BCAA diets (Table 1; Fig. 4A). Back transformed, the meta-analytic estimates suggest that food and energy intake in high dietary BCAA groups is typically 94% of that in low BCAA groups. Total heterogeneity was very high for all traits (*I*^2^_Total_ = 90–100%; Table 1), and low to moderate amounts of this variation was estimated to be from differences at the experimental level (*I*^2^_Experiment_ = 16–47%; Table 1).

For the effects of increased BCAAs on body mass, the amount of BCAAs in the reference diet was the moderator favoured by AIC (Additional File 1: Table S2). Where diets were low in BCAAs, increasing BCAAs resulted in increased body weight. However, for diets already high in BCAAs, further increases in BCAAs did not affect body mass (Fig. 4B). The AIC-favoured model for percentage fat mass included the magnitude of the increase in BCAAs between diets and the protein to carbohydrate ratio of the reference diet. On a low protein, high carbohydrate diet, relatively large increases of BCAAs had little effect on fat mass, whereas on high protein to carbohydrate diets, small increases in BCAAs were predicted to result in greater adiposity (Fig. 4C). Finally, the change in ratio of BCAAs to non-BCAAs was the best nutritional predictor of the effect of dietary BCAAs on both food and energy intake. Where the experimental diet had a lower ratio of BCAAs to non-BCAAs than the control diet, BCAAs resulted in elevated intake (Fig. 4D, E). However, where the experimental diet had a higher ratio of BCAAs to non-BCAAs than the control diet, food/energy intake was depressed (Fig. 4D, E).

Body weight was moderated by species, whereby effects sizes were slightly larger for rats than mice (Additional File 1: Table S3). The duration of exposure did not moderate the effect of dietary BCAAs on the intake or body composition (Additional File 1: Table S3). Egger’s regression suggested possible publication bias for percent fat mass and energy intake, although trim and fill analysis for these traits suggested no missing studies (Additional File 1: Table S4).

Together, these findings suggest that the effects of dietary BCAAs on body mass, composition and food intake are complex. Any effects are dependent on the dietary context in which BCAAs are elevated and any concomitant changes in other amino acids.

## Discussion

Here, we use multidimensional nutritional modelling, together with established techniques in meta-analysis and meta-regression, to disentangle the complex relationship between diet, BCAAs and metabolic health. The first issue we addressed was the relationship between dietary BCAAs and blood levels of BCAAs. Overall, there was a positive association between dietary and blood levels of BCAAs in both the fasting and fed state, supporting the notion that circulating BCAA levels are likely to reflect long-term protein intake [1, 7, 10, 17]. The relationship, however, is more nuanced. In animals restricted to diets containing different amounts of BCAAs, there was a curvilinear relationship between dietary content of BCAAs and the blood levels of BCAAs, a finding consistent with our previous experimental work. When the dietary background level of BCAAs was lower than standard mouse chow (0.52 kJ/g), increasing dietary BCAAs resulted in elevated blood levels of BCAA. At higher levels dietary BCAA, however, adding more to the diet had little effect. We have previously seen a plateau in circulating BCAAs when blood levels reach about 40 μg/mL [10, 22], occurring consistently when either total dietary protein content or dietary BCAA levels are increased beyond a point. This relationship reflects the network of mechanisms that influence BCAA levels. Blood levels of BCAAs are primarily regulated by BCKDH, a mitochondrial enzyme complex found in the liver and muscle that catabolizes the ketoacid metabolites of BCAAs. Because insulin and BCAAs both activate BCKDH which acts to reduce BCAA levels [17], the mechanism for the plateau in BCAA blood levels when dietary content is high may be explained by a compensatory increase in BCKDH activation. This plateau occurs once food content of BCAA is greater than in standard diets and when blood levels of BCAA are about 40 mg/L [10, 22], suggesting that in these conditions, the catabolic capacity of BCKDH cannot be further downregulated.

Another mechanism by which an animal can regulate blood levels of BCAAs is by altering dietary intake. As essential amino acids, BCAAs are primarily acquired through dietary sources. In our meta-analysis, animals only had access to a single diet; therefore, the only option for increasing or decreasing BCAA intake is by changing feeding behaviour to consume more, or less food. Evidence for this response was apparent in the analysis of the relationship between food intake and dietary BCAAs. Animals on diets high in BCAAs ate 94% of the amount eaten by animals on low BCAA diets. However, the effect of BCAAs food intake that we observe is small, even in these experimental animals where dietary BCAA levels are often dramatically manipulated (e.g. ranging from 20 to 200% of standard amounts of BCAA [10]). The impact of BCAAs on food intake is, however, complex. While the general trend showed that BCAAs reduced food intake of animals on diets with high BCAA, low non-BCAA ratios, it is important to note that many studies did not experimentally control for protein content when manipulating BCAA levels. It remains uncertain whether this reduction in food intake is attributable to the satiating effect of increasing total dietary protein.

While high amounts of dietary protein can suppress food intake and protein intake is prioritized over intake of fat and carbohydrates [23], the role of individual amino acids and their mixtures on protein appetite and food intake is complex and not yet fully understood. We found an effect of dietary BCAAs on food intake consistent with animals having the capacity to regulate food intake according to BCAA content; however, the effect is small and is likely confounded by the overall total protein content and balance of amino acids. Imbalance of amino acids is also known to influence feeding behaviour, with the effect of suppressing or increasing food intake dependent on the nature of the manipulation. For example, diets extremely deficient or devoid in one or more essential amino acids result in food aversion [24]. However, when the deficiency is small enough to be leveraged by compensatory feeding, hyperphagia is observed [25]. When compared to control groups, reducing dietary levels of single amino acids such as methionine, threonine or isoleucine [2, 26, 27] or groups of amino acids such as essential amino acids or the BCAAs [4, 5, 27] sufficiently increases food intake. In addition to dietary availability, the interaction between amino acids in circulation can regulate food intake by influencing whether the amino acid precursors necessary for neurotransmitter production are transported across the blood-brain barrier in sufficient quantities. A recent example showed that a diet high in BCAAs but low in tryptophan reduces uptake of tryptophan into the brain by competing for transport across the blood-brain barrier by the LAT1 amino acid transporter [10]. As tryptophan is the sole precursor for serotonin synthesis, a neurotransmitter involved in the control of food intake [28], reduced levels in the brain led to lower brain serotonin levels, greatly increased food intake, obesity and shortened lifespan. All these effects occurred without activation of canonical ageing pathways such as MTOR and IGF1 [10]. While this increase in food intake on high BCAA diets appear at odds with the findings of this meta-analysis, this effect may be explained by the interaction between dietary BCAAs and the total protein content of the diet. Many studies that supplement dietary BCAAs also increase the total protein content of the diet, an effect which will have important implications for promoting satiety. Solon-Biet et al 2019, however, use a unique design where BCAAs levels were doubled compared to the control group, while keeping total protein content constant. In experiments where this is not controlled, the effect of total dietary protein is likely to dominate any effect on appetite of dietary BCAAs.

What are the implications for human studies of the finding of this meta-analysis and the curvilinear relationship between dietary BCAAs and blood levels of BCAAs? First, it must be emphasized that these animal studies involved restriction to a single diet. Humans, on the other hand, have access to multiple foods with different contents of BCAAs, and other components such as tryptophan which can interact with BCAA to influence appetite. Second, unlike human studies, animal studies are undertaken with homogeneous genotypes and environments. Humans often have conditions and diseases unrelated to BCAA intake, but which may influence BCAA levels via their impact on various anabolic (insulin, IGF-1, GH) and catabolic (TNFα, cortisol, catecholamines, glucagon, inflammatory cytokines) factors that influence BCKDH activity. Even so, we predict that the curvilinear relationship between dietary BCAA and BCAA blood levels seen in animals will be apparent in human populations because it is a consequence of regulatory networks shared with humans. Thus, for human studies, we predict that there will the strongest association between individual dietary and blood levels in populations/groups with comparatively low levels of dietary BCAAs. While a positive correlation might be statistically significant over an entire range of blood levels and intakes, this may misrepresent the underlying curvilinear nature of the relationship. It must be noted, however, that BCAA levels are tightly regulated in the fasting period, so it is not simply a case of more dietary BCAAs entering the blood and increasing BCAA levels. If an association was found between dietary BCAAs and blood levels (when the dietary BCAAs are high), this may be explained by an indirect or confounding association that impacts on the regulatory network—in particular, BCKDH. For example, people with obesity may consume a diet with higher amounts of BCAAs but also have insulin resistance which impairs BCAA catabolism [19].

Here, we studied the relationship between blood levels of BCAAs and dietary BCAAs, but not total dietary protein. A weak association between dietary protein and blood levels of BCAAs has been reported in humans, and stronger associations in animal studies where protein intake and content can be strictly controlled [7, 29]. Although BCAAs are only found in dietary protein, the amount of BCAAs varies substantially depending on the source and type of protein, which makes evaluating any association more uncertain.

The second question we addressed with this meta-analysis was whether there are effects of dietary BCAAs on glucose metabolism, and if so, are these moderated by nutrient background? There were four metabolic outcomes assessed (insulin, glucose, glucose AUC and HOMA). Only glucose AUC had a significant overall association with dietary BCAAs, but not any other traits related to glucose homeostasis. In human studies, it has usually been reported that there is a strong association between dietary BCAAs and /or blood levels of BCAAs with impaired insulin and glucose metabolism, metabolic syndrome and diabetes [13, 19]. Although ascertaining the direction of causality in epidemiological studies is difficult, the most widely accepted conclusion is that elevated BCAAs are a consequence of insulin-resistant states—rather than elevated BCAAs contributing directly to insulin and glucose dysmetabolism, although there is evidence supporting both hypotheses [19]. The results of our meta-analysis are consistent with that interpretation. That is, we found in otherwise healthy animals (i.e. not obese or diabetic) on diets with sufficient levels of BCAAs/protein, changes in dietary BCAAs alone were not associated with overall significant metabolic disturbance. Model fitting, however, showed that these results are more nuanced and can be influenced by background nutrition. For glucose AUC, we found that the largest effects of dietary BCAAs occurred when there was a simultaneous increase in non-BCAA content (i.e. increasing protein content), a finding consistent with studies in humans where it has been shown that people not-subject to protein restriction have higher fasting blood glucose [5]. Although these results are complicated, the unifying theme is that when increased dietary BCAAs reflect increasing dietary protein, there is an increased association with glucose dysmetabolism. An association between excess dietary protein, particularly from animal sources, and cardiometabolic disorders has been widely reported [22, 30]. Thus, any association between BCAA and metabolic disease is more likely to be a result of BCAA being a biomarker for the amount and type of dietary protein, rather than being an independent risk factor.

Important outstanding questions raised by our study are (1) how quickly the effects of dietary protein/BCAAs on glucose metabolism take hold and (2) the degree to which any effects are reversible. On the first question, it seems unlikely that all outcomes respond similarly quickly to dietary BCAAs, yet our analyses detected few moderating effects of study duration. However, it is important to point out that our search and analysis did not explicitly target longitudinal experiments on the effects of dietary BCAAs. Regarding reversibility, this question requires examination of the responses to a diet switching experiments, which was also beyond the scope of the current synthesis. Nonetheless, some such studies have been performed. For example, Cummings et al. [8] found that reducing BCAAs and total amino acids, after animals had been exposed to a ‘western diet’ reduced fat mass and glucose AUC implying a degree of plasticity. However, Hahn et al. [31] found that a late-life switch to dietary restriction, which involves a reduction of all nutrients in the diet (including amino-acids), did not result in the expected improvements in survival, implying irreparable damage from the preceding diet. The GFN-based meta-regression approach that we present allows the user to identify the key nutritional dimensions of major effect, and thus may help to unify the results of different diet-switch experiments.

Finally, we addressed the issue of body composition and BCAAs. Overall, dietary BCAAs were not associated with body mass or body fat in this meta-analysis. However, there were associations when the underlying diet was considered. Increased BCAAs were associated with increased bodyweight when the background diet was low in BCAAs. This is likely a result of the relationship we found with food intake, where BCAAs were associated with increased food intake when the background diet was low in BCAAs, reflecting behavioural mechanisms of animals to reach intake targets of limiting nutrients [20]. It is important to note, however, that the balance of amino acids in the diet, in addition to macronutrient background, may exert different effects on food intake. For example, reducing levels of other specific amino acids such as tryptophan, while simultaneously increasing BCAAs may impair central appetite signalling mechanisms and promote hyperphagia [10]. Our meta-analysis also found that increased dietary BCAAs were associated with elevated body fat when the diet was high in protein and low in carbohydrates. This is consistent with amino acid biochemistry whereby excess amino acids above those required for protein synthesis can either be utilized via gluconeogenesis or ketogenesis for energy production or indirectly via acetyl coA converted to fat and glycogen [1, 14].

## Conclusion

This meta-analysis found that there was a curvilinear relationship between dietary BCAAs and blood levels of BCAAs, a finding consistent in both the fasting and fed state. It is important to note that these studies were undertaken in animal on restricted diets, and therefore, we must be cautious about extrapolating this finding to human data. We predict, however, that given shared regulatory mechanisms with humans, the curvilinear relationship between dietary BCAA and blood levels will be apparent in human populations. We also found that the relationship between dietary BCAAs and phenotypic outcomes (glucose and insulin, body composition and food intake) is complex and dependent on the underlying diet. This is an important finding for any study of dietary components and phenotypic outcomes because it emphasizes that diet is a complex mixture whereby each nutrient cannot be considered in isolation.

## Methods

### Search strategy and inclusion criteria

The methodology of this systematic review was pre-specified in a protocol and followed the guidelines of the Systematic Review Centre for Laboratory Animal [SYRCLE [32];]. A literature search was conducted in the databases Web of Science, Scopus, EMBASE and MEDLINE, as well as the specific journal Nature Metabolism, which was not indexed by those databases at the time. Keywords and search criteria were formulated, and are reported, using the guidelines in the PRISMA (Preferred Reporting Items for Systematic Reviews and Meta-Analyses) statement. The search was designed around the primary components ‘diet’, ‘branched chain amino acids’ and ‘rodents’ (for a full list of keywords for each database, see Additional File 2) and was last updated on 1/10/2019.

Screening of studies consisted of two phases. The first phase was based on title and abstract screening, and the second phase was based on a full-text screening. Two researchers independently conducted the first abstract and title screening phase, which was where 98% of studies were excluded. To be accepted for analysis the studies had to meet all of following inclusion criteria:

- We were able to obtain a copy of the full paper.
- The paper was written in English.
- An experimental mouse or rat study. Data was limited to healthy, non-gestating/lactating mice and rats who were free to move but not explicitly exercised. Studies on mutant/knockdown animals were excluded.
- Studies in which a dietary treatment involving an altered amount of BCAAs compared to a control group was administered. The BCAA increase/decrease must be achieved by altering the dietary complex (not via injection, or in water).
- Data must not come from diets that had less than 5% total energy from protein, as these diets were deemed outside of the envelope of nutritional space that can support rodent life or from a diet that the authors specifically designed to be ‘insufficient’ in terms of dietary protein/amino acid.
- The dietary treatment must be chronic, i.e. longer than 1 day.
- The study must report one or more of the outcome measures that quantifies circulating BCAA levels or cardio-metabolic health, as given in Table 1.
- The study must report the mean, sample size and preferably a measure of variability (e.g. standard deviation (SD)) for the outcome of interest (missing SDs were handled via multiple imputation).
- The study must report the composition of the diet, such that we were able to derive the energy density of the diet, and the percentage energy coming from macronutrients and BCAAs.

Studies were excluded at whichever phase they first were deemed to have violated any criteria, typically, though, assessment of criteria (e) through (i) required assessment of the full text (phase 2).

### Study characteristics and data extraction

The following data were extracted/derived from any included studies: bibliographic data, the rodent species, the duration of the treatment, diet composition in terms of percentage energy and overall energy density, whether animals were fasted prior to sampling, and the mean, sample size and SD of the outcome measures given in Table 1 (where necessary SD was derived from the standard error/confidence interval, although missing SDs were allowed and handled via multiple imputation; see below). Where necessary, energy content for protein/amino acids were calculated at 17 kJ/g, carbohydrates at 17 kJ/g and fat at 38 kJ/g. All data extraction was double checked by a second researcher.

Data were extracted from text or tables, and from graphs using the software GraphClick. When the group sizes were reported as a range, the midpoint was used and rounded up if not a whole number. Where outcome measures are reported as median and range, we estimated the mean and SD following Hozo et al. [33]. If data were reported over multiple time points, the longest duration for which concurrent data were available were used. For 6 studies we derived % body fat from total body mass and fat mass, in which case we assumed a strong correlation (*r* = 0.8) for the propagation of variation based on data from Solon-Biet et al. [22].

Leading investigators of studies or commercial providers of diets were contacted in cases where there was missing data. If the data were irretrievable the study was not included (those contacted needed to reply within two weeks of request via email).

### Effect sizes

All analyses were performed in the statistical programming environment R V4.1.0 [34]. Our effect size of use was the log response ratio (lnRR) sometimes called the ratio of means (ROM) and corresponds to the natural logarithm of ratio of the two means. We calculated lnRR such that positive values indicate a greater mean in the dietary group with greater (energy coming from) BCAAs, and negative values the opposite. Effect sizes and sampling variances were calculated using the ‘escalc’ function in the package *metafor* [35].

We calculated all pairwise comparisons within an experiment, for example in a study with 3 diet groups, there are 3 unique pairwise comparisons (A vs B, A vs C and B vs C), and thus, we calculated 3 lnRRs. This inevitably leads to covariance among effect sizes from the same experiments, termed ‘stochastic dependency’ [36]. Any such covariance was estimated following Lajeunesse [37], and the associated variance-covariance matrix was accounted for in any analyses [38]. In the event that such matrices were non-positive definite (a requirement for model fitting), a matrix bending procedure was employed (‘make.positive.definite’, in the *corpcor* package [39];).

Where authors had chosen to report their results as separate experiments (e.g. dietary interventional applied to different age classes), we treated them as such, and thus, single publications could contain multiple experiments. However, if authors split their results due to different diet composition (e.g. on different background levels of protein), we treated these as single experiments, with differences in diet composition used to determine the relative BCAA content of the diets. Potential moderating effects of other dietary factors were then explored using meta-regression (see below).

In the event that SDs were missing, we employed multiple imputation [40]. Imputation was performed on the log scale using the log mean as a predictor, with 20 replicate imputations. The whole set of analyses were applied to each imputed dataset with results pooled following DB Rubin [41]. The ‘mice’ function in the package *mice* [42] was used to impute missing SDs.

### Meta-analyses and meta-regression

Effect sizes for each health-related outcome were analysed separately. For each outcome, we began by fitting a multi-level meta-analysis (MLMA) which included the lnRR as the outcome and a variance-covariance matrix for the sampling variance. A random effect for the experimental unit (as there can be several effect sizes from a single experiment) from which the effect size came was included in all models. This first model estimated the overall effect size for the outcome of interest (in places log effect sizes are back transformed to raw ratios to aid in interpretation), its statistical significance (based on whether a 95% CI spans zero) and the degree of heterogeneity. For heterogeneity, we report the variance components as estimated by meta-analysis and from which we derive *I*^2^, which corresponds to the percentage of variation among effect sizes than cannot be attributed to sampling variation; 25, 50 and 75% were interpreted loosely as low, moderate and high heterogeneity [43]. As well as total *I*^2^, we partitioned *I*^2^ in to that explained by experimental ID following Nakagawa and Santos [44]. All models were implemented using the ‘rma.mv’ function in *metafor*, with terms estimated by restricted maximum likelihood (REML).

To try and understand the cause of heterogeneity (i.e. variation among reported effects), we used multi-level meta-regression (MLMR). To explore how nutritional aspects of experiments influenced the observed effect sizes, we fitted a series of MLMRs with different nutritional moderators, and selected among them based on Akaike information criterion (AIC [45];). Ranked alongside nutritional MLMRs was the equivalent MLMA, which served as a null model allowing for the possibility that none of the nutritional factors explored moderated the effect size. Models with the lowest AIC were favoured. In the event that models had AIC scores within 2 points of one another the simplest model (i.e. fewest parameters) was selected. We implemented a linear and non-linear variant of each nutritional moderator (providing that we had at least 10 effect sizes per parameter in the model). Non-linear models were fitted using basis splines of nutritional predictors using the ‘bs’ function in the *splines* package in *base* R (df=3). We explored a large number of nutritional predictors including the amount of BCAAs/total protein in the control diet, differences in BCAAs/protein between diets, interactions between these factors and the ratio of the protein to carbohydrate in the control diet. A complete list of the nutritional moderators explored for each outcome and their interpretation is given in the supplementary materials. Where data allowed, we also tested whether the effect size was predicted by the species (mouse or rat) and the duration of the dietary exposure (duration was log transformed to account for likely non-linear effect of exposure duration). For measures of circulating plasma BCAA levels and glucose metabolism we evaluated whether being fasted prior to sampling affected the effect size.

To visualize overall meta-analysis results, we use orchard plots [46]. To visualize the results of univariate meta-regressions involving numeric predictors, we use bubble plots. The results of multi-dimensional MLMR were visualized in multi-dimensional nutrient space using the surface-based approach common in the geometric framework for nutrition (GFN) [20]. Surfaces were coloured such that blue indicates a negative effect size, red positive and green a zero effect size at that point in the nutrient space. For all surfaces, a 95% CI was generated for each point in the nutrient space as the effect size at that point ± 1.96 × SE; where these CIs do not span zero the point on the surface was considered as differing significantly from zero (i.e. there is a statistically significant effect-size at this point in the nutrient space).

To assess potential effects of publication bias, we applied Egger’s regression to the residuals of the meta-analytic model for each trait using the ‘regtest’ function in *metafor*. In the event that Egger’s regression indicated significant asymmetry in the meta-analytic residuals of a trait, we applied a trim and fill test (‘trimfill’ in *metafor*) to estimate the number of missing studies and the effect of the inclusion of missing studies on the overall meta-analytic mean reported. Where imputation was used to estimate missing SDs, multiple instances of the publication bias tests were implemented with the results averaged.

## Supplementary Information

**Additional File 1.** Tables S1. Studies included in the analysis of each trait. Tables S2. Relative model fit of nutritional meta-regressions, based on Akaike Information Criterion (AIC). Tables S3. Model coefficients meta-regressions of species and fasting for each trait. Tables S4. Results of publication bias tests for each trait. Tables S5. Model coefficients for AIC favored meta-regression for each trait as shown intake S1. Table S6. Details of articles analyzed.

**Additional File 2.** Detailed methods.

## Data Availability

All code and data for the analyses contained herein is available at https://github.com/AlistairMcNairSenior/BCAAs_Meta_Analysis
