# Supplementary material for: Meta-analysis links dietary branched-chain amino acids to metabolic health in rodents
Source: BMC Biol. 2022 Jan 14;20:19. doi: 10.1186/s12915-021-01201-2 (PMC8760763; doi:10.1186/s12915-021-01201-2)
Supplement: Supplementary file 1 — Additional File 1. Tables S1. Studies included in the analysis of each trait. Tables S2. Relative model fit of nutritional meta-regressions, based on Akaike Information Criterion (AIC). Tables S3. Model coefficients meta-regressions of species and fasting for each trait. Tables S4. Results of publication bias tests for each trait. Tables S5. Model coefficients for AIC favored meta-regression for each trait as shown intake S1. Table S6. Details of articles analyzed. [file 12915_2021_1201_MOESM1_ESM.docx]

**Table S1.**

Studies included in the analysis of each trait (1 = included). See Table S6 for detailed references for each article.

| Article | Circulating BCAAs | Circulating Isoleucine | Circulating Leucine | Circulating Valine | Glucose AUC | Plasma Glucose | Plasma Insulin | HOMA | Mass | Fat % | Intake | Energy Intake |
| --- | --- | --- | --- | --- | --- | --- | --- | --- | --- | --- | --- | --- |
| [1] |  |  |  |  |  | 1 | 1 |  | 1 |  | 1 | 1 |
| [2] |  |  | 1 |  |  | 1 | 1 |  | 1 |  |  |  |
| [3] |  | 1 | 1 | 1 |  |  |  |  | 1 |  | 1 | 1 |
| [4] | 1 | 1 | 1 | 1 |  |  |  |  | 1 |  | 1 | 1 |
| [5] |  |  |  |  |  |  |  |  | 1 |  | 1 | 1 |
| [6] |  | 1 | 1 | 1 |  |  |  |  | 1 |  | 1 | 1 |
| [7] | 1 |  |  |  |  | 1 | 1 |  | 1 |  | 1 | 1 |
| [8] |  |  |  |  |  | 1 | 1 |  | 1 |  | 1 | 1 |
| [9] |  | 1 | 1 | 1 |  |  |  |  | 1 |  | 1 | 1 |
| [10] |  | 1 | 1 | 1 |  | 1 | 1 |  | 1 |  | 1 | 1 |
| [11] |  |  |  |  |  |  |  |  | 1 |  |  |  |
| [12] |  |  |  |  |  |  |  |  | 1 |  | 1 | 1 |
| [13] |  |  |  |  |  |  |  |  | 1 |  | 1 | 1 |
| [14] |  |  | 1 |  |  |  |  |  | 1 |  |  |  |
| [15] |  |  |  |  |  |  |  |  | 1 |  |  |  |
| [16] |  |  |  |  |  |  |  |  | 1 |  | 1 | 1 |
| [17] |  |  |  |  |  | 1 | 1 | 1 | 1 |  | 1 | 1 |
| [18] |  |  |  |  |  | 1 | 1 | 1 | 1 |  | 1 | 1 |
| [19] |  | 1 | 1 | 1 |  | 1 |  |  | 1 |  | 1 | 1 |
| [20] |  | 1 | 1 | 1 |  | 1 | 1 |  | 1 |  | 1 | 1 |
| [21] |  | 1 | 1 | 1 |  |  |  |  | 1 |  | 1 | 1 |
| [22] |  |  |  |  |  |  |  |  | 1 |  | 1 | 1 |
| [23] |  | 1 | 1 | 1 |  |  |  |  | 1 |  | 1 | 1 |
| [24] |  |  |  |  | 1 | 1 | 1 | 1 | 1 | 1 | 1 | 1 |
| [25] |  |  |  |  |  | 1 | 1 |  | 1 |  |  |  |
| [26] |  |  |  |  |  |  |  |  | 1 |  |  |  |
| [27] |  |  |  |  | 1 |  | 1 | 1 | 1 |  |  |  |
| [28] |  |  |  |  |  | 1 | 1 |  | 1 |  |  |  |
| [29] |  |  |  |  | 1 | 1 | 1 |  | 1 |  |  |  |
| [30] |  | 1 | 1 | 1 |  | 1 | 1 |  | 1 |  | 1 | 1 |
| [31] |  | 1 | 1 | 1 |  | 1 | 1 |  | 1 | 1 | 1 | 1 |
| [32] |  | 1 | 1 | 1 |  | 1 | 1 |  | 1 | 1 | 1 | 1 |
| [33] |  |  |  |  | 1 | 1 |  |  |  |  |  |  |
| [34] |  |  |  |  | 1 | 1 | 1 |  | 1 |  |  |  |
| [35] |  |  |  |  |  |  |  |  | 1 |  | 1 | 1 |
| [36] |  |  |  |  |  |  |  |  | 1 |  | 1 | 1 |
| [37] |  |  |  |  |  |  |  |  | 1 |  |  |  |
| [38] |  |  |  |  |  |  |  |  | 1 |  | 1 | 1 |
| [39] |  |  |  |  |  |  |  |  | 1 |  | 1 | 1 |
| [40] |  |  |  |  |  |  |  |  | 1 |  | 1 | 1 |
| [41] |  |  |  |  |  |  |  |  | 1 |  | 1 | 1 |
| [42] |  |  |  |  |  |  |  |  | 1 |  | 1 | 1 |
| [43] |  | 1 | 1 | 1 |  |  |  |  | 1 |  | 1 | 1 |
| [44] |  |  |  |  |  |  |  |  |  |  | 1 | 1 |
| [45] |  |  |  |  |  |  |  |  | 1 |  | 1 | 1 |
| [46] |  |  |  |  |  | 1 | 1 | 1 | 1 |  | 1 | 1 |
| [47] |  |  |  |  |  | 1 | 1 |  | 1 | 1 | 1 | 1 |
| [48] |  | 1 | 1 | 1 | 1 | 1 | 1 | 1 |  |  |  |  |
| [49] |  | 1 | 1 | 1 |  | 1 |  |  | 1 |  | 1 | 1 |
| [50] |  | 1 | 1 | 1 |  |  | 1 |  | 1 |  |  |  |
| [51] |  |  |  |  |  |  |  |  | 1 |  | 1 | 1 |
| [52] |  | 1 | 1 | 1 |  |  |  |  | 1 | 1 |  |  |
| [53] |  |  |  |  |  | 1 |  |  | 1 |  | 1 | 1 |
| [54] |  | 1 | 1 | 1 |  |  |  |  | 1 |  |  |  |
| [55] |  |  |  |  |  |  |  |  | 1 |  |  |  |
| [56] |  |  |  |  |  |  |  |  | 1 |  |  |  |
| [57] |  |  |  |  | 1 | 1 | 1 |  | 1 |  | 1 | 1 |
| [58] |  |  |  |  |  |  |  |  |  |  | 1 | 1 |
| [59] |  |  |  |  | 1 | 1 |  |  | 1 | 1 | 1 | 1 |
| [60] |  |  |  |  |  |  |  |  | 1 |  | 1 | 1 |
| [61] |  | 1 | 1 | 1 |  |  |  |  | 1 |  |  |  |
| [62] |  |  |  |  |  |  |  |  | 1 |  | 1 | 1 |
| [63] |  | 1 | 1 | 1 |  | 1 | 1 |  | 1 |  | 1 | 1 |
| [64] |  | 1 | 1 | 1 |  | 1 | 1 |  | 1 | 1 | 1 | 1 |
| [65] |  |  |  |  | 1 | 1 | 1 | 1 | 1 |  | 1 | 1 |
| [66] |  |  |  |  |  |  |  |  | 1 | 1 |  |  |
| [67] |  | 1 | 1 | 1 |  |  |  |  | 1 |  | 1 | 1 |
| [68] |  | 1 | 1 | 1 |  |  |  |  | 1 |  | 1 | 1 |
| [69] |  | 1 | 1 | 1 |  |  |  |  | 1 |  | 1 | 1 |
| [70] |  | 1 | 1 |  |  |  |  |  | 1 |  | 1 | 1 |
| [71] |  |  |  |  |  |  |  |  | 1 |  |  |  |
| [72] |  |  |  |  |  |  |  |  | 1 |  | 1 | 1 |
| [73] |  |  |  |  |  |  |  |  | 1 |  | 1 | 1 |
| [74] |  |  |  | 1 |  |  |  |  | 1 |  | 1 | 1 |
| [75] |  |  |  |  |  |  |  |  | 1 |  | 1 | 1 |
| [76] |  | 1 | 1 | 1 |  | 1 | 1 |  | 1 |  | 1 | 1 |
| [77] |  | 1 | 1 | 1 |  |  |  |  | 1 |  | 1 | 1 |
| [78] |  | 1 | 1 | 1 |  |  |  |  |  |  |  |  |
| [79] | 1 | 1 | 1 | 1 | 1 |  | 1 | 1 | 1 | 1 | 1 | 1 |
| [80] | 1 | 1 | 1 | 1 |  |  | 1 |  | 1 | 1 | 1 | 1 |
| [81] |  |  |  |  |  |  |  |  | 1 |  | 1 | 1 |
| [82] |  |  |  |  |  |  |  |  | 1 |  |  |  |
| [83] |  |  |  |  |  |  |  |  | 1 |  |  |  |
| [84] |  |  |  |  |  |  |  |  | 1 |  |  |  |
| [85] |  |  |  |  |  |  |  |  | 1 |  | 1 | 1 |
| [86] |  |  |  |  |  |  |  |  | 1 |  |  |  |
| [87] |  | 1 | 1 | 1 |  | 1 | 1 | 1 | 1 | 1 | 1 | 1 |
| [88] |  | 1 | 1 | 1 |  | 1 | 1 | 1 | 1 | 1 | 1 | 1 |
| [89] |  | 1 | 1 | 1 |  | 1 |  |  | 1 |  |  |  |
| [90] |  |  |  |  |  |  |  |  | 1 |  | 1 | 1 |
| [91] |  |  |  |  |  |  |  |  | 1 |  | 1 | 1 |
| [92] | 1 | 1 | 1 | 1 |  |  |  |  | 1 |  | 1 | 1 |
| [93] |  |  |  |  |  |  |  |  | 1 |  | 1 | 1 |

**Table S2**

Relative model fit of nutritional meta-regressions, based on Akaike Information Criterion (AIC). DF the number of model coefficients, Δ AIC is differences in AIC relative to lowest AIC, BS indicates a basis spline, * indicates an interaction between terms, diff indicates the difference between experimental and control diets and MA indicates the meta-analysis (i.e. null model).

| Trait | Moderator | DF | AIC | Δ AIC |
| --- | --- | --- | --- | --- |
| Plasma BCAAs | BS(Diet_BCAA_kJ_g) | 4 | 260 | 0 |
|  | Diet_BCAA_kJ_g | 2 | 273 | 13 |
|  | Diet_BCAA_kJ_g * diff_BCAA | 4 | 276 | 16 |
|  | BS(Diet_BCAA_kJ_g) * BS(diff_BCAA) | 10 | 300 | 39 |
|  | BS(diff_BCAA) | 4 | 307 | 46 |
|  | diff_BCAA | 2 | 310 | 49 |
|  | Diet_P_kJ_g | 2 | 314 | 53 |
|  | BS(Diet_P_kJ_g) | 4 | 315 | 54 |
|  | Diet_P_kJ_g * diff_P | 4 | 316 | 55 |
|  | BS(diff_P) | 4 | 317 | 57 |
|  | diff_P | 2 | 318 | 57 |
|  | MA | 1 | 322 | 61 |
|  | BS(Diet_P_kJ_g) * BS(diff_P) | 10 | 331 | 70 |
| Plasma Isoleucine | Diet_Iso_kJ_g * diff_Iso | 4 | 564 | 0 |
|  | BS(Diet_Iso_kJ_g) * BS(diff_Iso) | 10 | 570 | 6 |
|  | diff_Iso | 2 | 588 | 24 |
|  | BS(diff_Iso) | 4 | 590 | 26 |
|  | Diet_Iso_kJ_g | 2 | 613 | 49 |
|  | BS(Diet_Iso_kJ_g) | 4 | 614 | 50 |
|  | Diet_P_kJ_g * diff_P | 4 | 631 | 67 |
|  | BS(diff_P) | 4 | 639 | 75 |
|  | BS(Diet_P_kJ_g) * BS(diff_P) | 10 | 640 | 77 |
|  | diff_P | 2 | 641 | 78 |
|  | BS(Diet_P_kJ_g) | 4 | 656 | 92 |
|  | Diet_P_kJ_g | 2 | 659 | 96 |
|  | MA | 1 | 691 | 128 |
| Plasma Leucine | Diet_Leu_kJ_g * diff_Leu | 4 | 320 | 0 |
|  | BS(Diet_Leu_kJ_g) * BS(diff_Leu) | 10 | 325 | 5 |
|  | BS(diff_Leu) | 4 | 334 | 14 |
|  | diff_Leu | 2 | 338 | 18 |
|  | BS(Diet_Leu_kJ_g) | 4 | 351 | 31 |
|  | Diet_Leu_kJ_g | 2 | 374 | 54 |
|  | Diet_P_kJ_g * diff_P | 4 | 391 | 71 |
|  | BS(Diet_P_kJ_g) * BS(diff_P) | 10 | 398 | 78 |
|  | BS(diff_P) | 4 | 409 | 89 |
|  | diff_P | 2 | 414 | 94 |
|  | BS(Diet_P_kJ_g) | 4 | 420 | 100 |
|  | Diet_P_kJ_g | 2 | 424 | 104 |
|  | MA | 1 | 487 | 167 |
| Plasma Valine | Diet_Val_kJ_g * diff_Val | 4 | 496 | 0 |
|  | BS(Diet_Val_kJ_g) | 4 | 498 | 2 |
|  | BS(Diet_Val_kJ_g) * BS(diff_Val) | 10 | 506 | 9 |
|  | Diet_Val_kJ_g | 2 | 510 | 13 |
|  | BS(diff_Val) | 4 | 532 | 35 |
|  | diff_Val | 2 | 538 | 41 |
|  | Diet_P_kJ_g * diff_P | 4 | 558 | 62 |
|  | diff_P | 2 | 562 | 65 |
|  | BS(diff_P) | 4 | 564 | 68 |
|  | Diet_P_kJ_g | 2 | 568 | 72 |
|  | BS(Diet_P_kJ_g) | 4 | 569 | 73 |
|  | BS(Diet_P_kJ_g) * BS(diff_P) | 10 | 577 | 81 |
|  | MA | 1 | 585 | 89 |
| Glucose AUC | diff_nonBCAA * diff_BCAA | 4 | 148 | 0 |
|  | BS(diff_BCAA) | 4 | 149 | 1 |
|  | Diet_BCAA_kJ_g * diff_BCAA | 4 | 151 | 4 |
|  | diff_BCAA * diff_Val | 4 | 152 | 4 |
|  | Diet_P_kJ_g * diff_BCAA | 4 | 152 | 4 |
|  | diff_BCAA * diff_Iso | 4 | 153 | 5 |
|  | diff_BCAA * diff_Leu | 4 | 153 | 5 |
|  | diff_BCAA | 2 | 158 | 11 |
|  | Diet_PC_kJ_g * diff_BCAA | 4 | 163 | 15 |
|  | BS(Diet_BCAA_kJ_g) | 4 | 185 | 38 |
|  | BS(Diet_P_kJ_g) | 4 | 189 | 41 |
|  | Diet_BCAA_kJ_g | 2 | 189 | 41 |
|  | Diet_P_kJ_g | 2 | 191 | 44 |
|  | BS(Diet_BCAA_kJ_g) * BS(diff_BCAA) | 10 | 196 | 49 |
|  | BS(Diet_P_kJ_g) * BS(diff_BCAA) | 10 | 198 | 50 |
|  | BS(Diet_PC_kJ_g) * BS(diff_BCAA) | 10 | 213 | 65 |
|  | BS(Diet_PC_kJ_g) | 4 | 226 | 79 |
|  | Diet_PC_kJ_g | 2 | 228 | 80 |
|  | MA | 1 | 230 | 82 |
|  | diff_BCAA.Non_BCAA_kJ_g | 2 | 232 | 84 |
|  | BS(diff_BCAA.Non_BCAA_kJ_g) | 4 | 236 | 88 |
| Plasma Glucose | MA | 1 | -184 | 0 |
|  | Diet_BCAA_kJ_g | 2 | -183 | 1 |
|  | Diet_PC_kJ_g | 2 | -183 | 2 |
|  | Diet_P_kJ_g | 2 | -181 | 3 |
|  | diff_BCAA.Non_BCAA_kJ_g | 2 | -180 | 4 |
|  | diff_BCAA | 2 | -180 | 4 |
|  | Diet_BCAA_kJ_g * diff_BCAA | 4 | -180 | 5 |
|  | BS(Diet_PC_kJ_g) | 4 | -177 | 7 |
|  | BS(Diet_BCAA_kJ_g) | 4 | -176 | 9 |
|  | Diet_PC_kJ_g * diff_BCAA | 4 | -175 | 9 |
|  | Diet_P_kJ_g * diff_BCAA | 4 | -175 | 9 |
|  | diff_BCAA * diff_Val | 4 | -175 | 10 |
|  | BS(Diet_P_kJ_g) | 4 | -175 | 10 |
|  | diff_BCAA * diff_Iso | 4 | -174 | 11 |
|  | diff_BCAA * diff_Leu | 4 | -173 | 11 |
|  | diff_nonBCAA * diff_BCAA | 4 | -173 | 11 |
|  | BS(diff_BCAA.Non_BCAA_kJ_g) | 4 | -172 | 12 |
|  | BS(diff_BCAA) | 4 | -171 | 13 |
|  | BS(Diet_BCAA_kJ_g) * BS(diff_BCAA) | 10 | -159 | 25 |
|  | BS(Diet_P_kJ_g) * BS(diff_BCAA) | 10 | -154 | 31 |
|  | BS(diff_BCAA) * BS(diff_Iso) | 10 | -152 | 32 |
|  | BS(diff_BCAA) * BS(diff_Val) | 10 | -152 | 33 |
|  | BS(diff_nonBCAA) * BS(diff_BCAA) | 10 | -151 | 33 |
|  | BS(diff_BCAA) * BS(diff_Leu) | 10 | -149 | 35 |
|  | BS(Diet_PC_kJ_g) * BS(diff_BCAA) | 10 | -149 | 36 |
| Plasma Insulin | BS(Diet_PC_kJ_g) | 4 | 480 | 0 |
|  | BS(Diet_BCAA_kJ_g) | 4 | 500 | 20 |
|  | BS(Diet_P_kJ_g) | 4 | 508 | 27 |
|  | BS(Diet_PC_kJ_g) * BS(diff_BCAA) | 10 | 517 | 37 |
|  | Diet_PC_kJ_g * diff_BCAA | 4 | 536 | 55 |
|  | BS(Diet_P_kJ_g) * BS(diff_BCAA) | 10 | 540 | 60 |
|  | Diet_BCAA_kJ_g * diff_BCAA | 4 | 549 | 68 |
|  | BS(Diet_BCAA_kJ_g) * BS(diff_BCAA) | 10 | 550 | 69 |
|  | Diet_BCAA_kJ_g | 2 | 550 | 70 |
|  | Diet_PC_kJ_g | 2 | 551 | 70 |
|  | Diet_P_kJ_g * diff_BCAA | 4 | 556 | 76 |
|  | BS(diff_nonBCAA) * BS(diff_BCAA) | 10 | 557 | 76 |
|  | Diet_P_kJ_g | 2 | 557 | 76 |
|  | BS(diff_BCAA) * BS(diff_Leu) | 10 | 560 | 79 |
|  | BS(diff_BCAA) * BS(diff_Iso) | 10 | 560 | 79 |
|  | BS(diff_BCAA) | 4 | 563 | 82 |
|  | diff_BCAA * diff_Val | 4 | 563 | 82 |
|  | diff_BCAA | 2 | 563 | 83 |
|  | diff_BCAA * diff_Leu | 4 | 563 | 83 |
|  | diff_BCAA * diff_Iso | 4 | 563 | 83 |
|  | diff_nonBCAA * diff_BCAA | 4 | 565 | 84 |
|  | BS(diff_BCAA) * BS(diff_Val) | 10 | 565 | 84 |
|  | MA | 1 | 567 | 86 |
|  | diff_BCAA.Non_BCAA_kJ_g | 2 | 568 | 87 |
|  | BS(diff_BCAA.Non_BCAA_kJ_g) | 4 | 571 | 90 |
| HOMA | BS(Diet_P_kJ_g) | 4 | 504 | 0 |
|  | BS(Diet_BCAA_kJ_g) | 4 | 509 | 6 |
|  | BS(Diet_PC_kJ_g) | 4 | 536 | 32 |
|  | Diet_BCAA_kJ_g * diff_BCAA | 4 | 549 | 45 |
|  | Diet_BCAA_kJ_g | 2 | 553 | 49 |
|  | BS(Diet_P_kJ_g) * BS(diff_BCAA) | 10 | 557 | 53 |
|  | BS(Diet_BCAA_kJ_g) * BS(diff_BCAA) | 10 | 569 | 65 |
|  | BS(Diet_PC_kJ_g) * BS(diff_BCAA) | 10 | 570 | 66 |
|  | Diet_PC_kJ_g * diff_BCAA | 4 | 572 | 68 |
|  | Diet_P_kJ_g * diff_BCAA | 4 | 573 | 69 |
|  | Diet_P_kJ_g | 2 | 574 | 70 |
|  | BS(diff_BCAA) * BS(diff_Val) | 10 | 575 | 72 |
|  | BS(diff_BCAA) * BS(diff_Iso) | 10 | 577 | 73 |
|  | BS(diff_nonBCAA) * BS(diff_BCAA) | 10 | 577 | 73 |
|  | BS(diff_BCAA) * BS(diff_Leu) | 10 | 579 | 75 |
|  | diff_BCAA * diff_Val | 4 | 579 | 76 |
|  | diff_BCAA * diff_Iso | 4 | 580 | 76 |
|  | diff_BCAA * diff_Leu | 4 | 581 | 77 |
|  | BS(diff_BCAA) | 4 | 583 | 80 |
|  | diff_nonBCAA * diff_BCAA | 4 | 584 | 80 |
|  | diff_BCAA | 2 | 586 | 83 |
|  | Diet_PC_kJ_g | 2 | 591 | 87 |
|  | BS(diff_BCAA.Non_BCAA_kJ_g) | 4 | 591 | 87 |
|  | diff_BCAA.Non_BCAA_kJ_g | 2 | 595 | 91 |
|  | MA | 1 | 595 | 92 |
| Mass | BS(Diet_BCAA_kJ_g) | 4 | -873 | 0 |
|  | BS(Diet_PC_kJ_g) | 4 | -859 | 14 |
|  | BS(Diet_P_kJ_g) | 4 | -818 | 55 |
|  | Diet_P_kJ_g * diff_BCAA | 4 | -809 | 64 |
|  | BS(Diet_BCAA_kJ_g) * BS(diff_BCAA) | 10 | -807 | 65 |
|  | BS(Diet_PC_kJ_g) * BS(diff_BCAA) | 10 | -806 | 66 |
|  | Diet_BCAA_kJ_g * diff_BCAA | 4 | -802 | 71 |
|  | diff_BCAA.Non_BCAA_kJ_g | 2 | -800 | 73 |
|  | BS(diff_BCAA.Non_BCAA_kJ_g) | 4 | -795 | 77 |
|  | BS(Diet_P_kJ_g) * BS(diff_BCAA) | 10 | -791 | 81 |
|  | BS(diff_BCAA) * BS(diff_Val) | 10 | -790 | 83 |
|  | Diet_BCAA_kJ_g | 2 | -789 | 84 |
|  | Diet_P_kJ_g | 2 | -787 | 85 |
|  | Diet_PC_kJ_g | 2 | -781 | 91 |
|  | Diet_PC_kJ_g * diff_BCAA | 4 | -778 | 95 |
|  | diff_nonBCAA * diff_BCAA | 4 | -775 | 97 |
|  | MA | 1 | -774 | 99 |
|  | diff_BCAA | 2 | -774 | 99 |
|  | diff_BCAA * diff_Val | 4 | -771 | 102 |
|  | diff_BCAA * diff_Leu | 4 | -769 | 104 |
|  | BS(diff_BCAA) | 4 | -769 | 104 |
|  | diff_BCAA * diff_Iso | 4 | -768 | 105 |
|  | BS(diff_nonBCAA) * BS(diff_BCAA) | 10 | -765 | 108 |
|  | BS(diff_BCAA) * BS(diff_Leu) | 10 | -759 | 113 |
|  | BS(diff_BCAA) * BS(diff_Iso) | 10 | -754 | 118 |
| Percent Fat Mass | Diet_PC_kJ_g * diff_BCAA | 4 | 29 | 0 |
|  | BS(Diet_PC_kJ_g) | 4 | 50 | 20 |
|  | Diet_PC_kJ_g | 2 | 56 | 27 |
|  | BS(Diet_PC_kJ_g) * BS(diff_BCAA) | 10 | 63 | 33 |
|  | BS(Diet_P_kJ_g) | 4 | 98 | 69 |
|  | diff_BCAA | 2 | 99 | 70 |
|  | diff_nonBCAA * diff_BCAA | 4 | 101 | 72 |
|  | diff_BCAA * diff_Leu | 4 | 101 | 72 |
|  | diff_BCAA * diff_Val | 4 | 101 | 72 |
|  | diff_BCAA * diff_Iso | 4 | 102 | 73 |
|  | BS(diff_BCAA) | 4 | 102 | 73 |
|  | BS(Diet_BCAA_kJ_g) | 4 | 103 | 74 |
|  | Diet_P_kJ_g * diff_BCAA | 4 | 103 | 74 |
|  | MA | 1 | 104 | 74 |
|  | Diet_BCAA_kJ_g * diff_BCAA | 4 | 104 | 74 |
|  | diff_BCAA.Non_BCAA_kJ_g | 2 | 104 | 75 |
|  | Diet_BCAA_kJ_g | 2 | 105 | 76 |
|  | Diet_P_kJ_g | 2 | 106 | 76 |
|  | BS(diff_BCAA.Non_BCAA_kJ_g) | 4 | 107 | 78 |
|  | BS(Diet_P_kJ_g) * BS(diff_BCAA) | 10 | 108 | 79 |
|  | BS(Diet_BCAA_kJ_g) * BS(diff_BCAA) | 10 | 109 | 80 |
|  | BS(diff_BCAA) * BS(diff_Val) | 10 | 112 | 83 |
|  | BS(diff_BCAA) * BS(diff_Leu) | 10 | 112 | 83 |
|  | BS(diff_BCAA) * BS(diff_Iso) | 10 | 113 | 84 |
|  | BS(diff_nonBCAA) * BS(diff_BCAA) | 10 | 117 | 87 |
| Food Intake | BS(diff_BCAA.Non_BCAA_kJ_g) | 4 | -589 | 0 |
|  | Diet_PC_kJ_g * diff_BCAA | 4 | -538 | 50 |
|  | diff_BCAA.Non_BCAA_kJ_g | 2 | -516 | 73 |
|  | BS(diff_BCAA) * BS(diff_Val) | 10 | -515 | 74 |
|  | Diet_P_kJ_g * diff_BCAA | 4 | -512 | 77 |
|  | BS(Diet_PC_kJ_g) * BS(diff_BCAA) | 10 | -512 | 77 |
|  | diff_BCAA | 2 | -510 | 79 |
|  | diff_BCAA * diff_Leu | 4 | -510 | 79 |
|  | diff_BCAA * diff_Iso | 4 | -509 | 80 |
|  | BS(diff_BCAA) | 4 | -508 | 81 |
|  | diff_BCAA * diff_Val | 4 | -507 | 82 |
|  | Diet_BCAA_kJ_g * diff_BCAA | 4 | -505 | 84 |
|  | diff_nonBCAA * diff_BCAA | 4 | -505 | 84 |
|  | BS(diff_BCAA) * BS(diff_Iso) | 10 | -504 | 85 |
|  | BS(diff_BCAA) * BS(diff_Leu) | 10 | -502 | 87 |
|  | BS(diff_nonBCAA) * BS(diff_BCAA) | 10 | -499 | 90 |
|  | BS(Diet_BCAA_kJ_g) * BS(diff_BCAA) | 10 | -494 | 95 |
|  | BS(Diet_P_kJ_g) * BS(diff_BCAA) | 10 | -464 | 125 |
|  | BS(Diet_BCAA_kJ_g) | 4 | -444 | 144 |
|  | BS(Diet_PC_kJ_g) | 4 | -432 | 157 |
|  | Diet_BCAA_kJ_g | 2 | -425 | 164 |
|  | Diet_PC_kJ_g | 2 | -416 | 173 |
|  | MA | 1 | -412 | 177 |
|  | Diet_P_kJ_g | 2 | -411 | 178 |
|  | BS(Diet_P_kJ_g) | 4 | -409 | 180 |
| Energy Intake | BS(diff_BCAA.Non_BCAA_kJ_g) | 4 | -332 | 0 |
|  | diff_BCAA.Non_BCAA_kJ_g | 2 | -296 | 36 |
|  | Diet_PC_kJ_g * diff_BCAA | 4 | -268 | 64 |
|  | Diet_PC_kJ_g | 2 | -261 | 71 |
|  | BS(Diet_PC_kJ_g) | 4 | -256 | 77 |
|  | BS(Diet_PC_kJ_g) * BS(diff_BCAA) | 10 | -252 | 81 |
|  | Diet_BCAA_kJ_g * diff_BCAA | 4 | -240 | 92 |
|  | BS(Diet_P_kJ_g) | 4 | -238 | 94 |
|  | BS(Diet_P_kJ_g) * BS(diff_BCAA) | 10 | -238 | 94 |
|  | Diet_P_kJ_g * diff_BCAA | 4 | -237 | 95 |
|  | diff_BCAA | 2 | -237 | 95 |
|  | diff_BCAA * diff_Leu | 4 | -235 | 97 |
|  | diff_nonBCAA * diff_BCAA | 4 | -234 | 98 |
|  | diff_BCAA * diff_Val | 4 | -233 | 100 |
|  | BS(diff_BCAA) | 4 | -233 | 100 |
|  | diff_BCAA * diff_Iso | 4 | -231 | 101 |
|  | Diet_BCAA_kJ_g | 2 | -226 | 106 |
|  | BS(diff_nonBCAA) * BS(diff_BCAA) | 10 | -223 | 109 |
|  | MA | 1 | -222 | 110 |
|  | BS(Diet_BCAA_kJ_g) | 4 | -222 | 110 |
|  | BS(Diet_BCAA_kJ_g) * BS(diff_BCAA) | 10 | -222 | 111 |
|  | BS(diff_BCAA) * BS(diff_Val) | 10 | -221 | 111 |
|  | Diet_P_kJ_g | 2 | -221 | 111 |
|  | BS(diff_BCAA) * BS(diff_Leu) | 10 | -218 | 114 |
|  | BS(diff_BCAA) * BS(diff_Iso) | 10 | -218 | 114 |

**Table S3**

Model coefficients meta-regressions of species and fasting for each trait. LCL and UCL is the lower and upper 95% confidence limit. Where the LCL and UCL for the difference between groups excludes zero, the moderator is considered statistically significant, and is highlighted in bold.

| Trait | Model | Coefficient | Estimate | LCL | UCL |
| --- | --- | --- | --- | --- | --- |
| Plasma BCAA | Species | Mouse | 0.158 | -0.174 | 0.490 |
|  |  | Rat – Mouse | 0.265 | -0.207 | 0.736 |
|  | Time Fasted | Fasted | 0.204 | 0.032 | 0.376 |
|  |  | Fed – Fasted | 0.707 | -0.032 | 1.446 |
|  | Duration | Intercept | 0.418 | 0.031 | 0.806 |
|  |  | Weeks | -0.042 | -0.145 | 0.062 |
| Plasma Isoleucine | Species | Mouse | 0.236 | 0.022 | 0.449 |
|  |  | Rat – Mouse | -0.166 | -0.424 | 0.092 |
|  | Time Fasted | Fasted | 0.146 | -0.027 | 0.319 |
|  |  | Fed – Fasted | -0.045 | -0.286 | 0.195 |
|  | Duration | Intercept | 0.181 | 0.004 | 0.358 |
|  |  | Weeks | -0.037 | -0.121 | 0.047 |
| Plasma Leucine | Species | Mouse | 0.222 | 0.056 | 0.388 |
|  |  | Rat – Mouse | 0.142 | -0.059 | 0.343 |
|  | **Time Fasted** | **Fasted** | **0.219** | **0.096** | **0.342** |
|  |  | **Fed – Fasted** | **0.211** | **0.033** | **0.389** |
|  | **Duration** | **Intercept** | **0.423** | **0.287** | **0.560** |
|  |  | **Weeks** | **-0.064** | **-0.126** | **-0.002** |
| Plasma Valine | Species | Mouse | 0.262 | 0.065 | 0.458 |
|  |  | Rat – Mouse | -0.100 | -0.343 | 0.143 |
|  | Time Fasted | Fasted | 0.104 | -0.042 | 0.251 |
|  |  | Fed – Fasted | 0.206 | -0.010 | 0.421 |
|  | Duration | Intercept | 0.275 | 0.111 | 0.439 |
|  |  | Weeks | -0.045 | -0.119 | 0.028 |
| Glucose AUC | Species | Mouse | 0.243 | 0.123 | 0.362 |
|  |  | Rat – Mouse | -0.253 | -0.573 | 0.068 |
|  | Time Fasted | Fasted | 0.245 | 0.127 | 0.363 |
|  |  | Fed – Fasted | -0.297 | -0.633 | 0.040 |
|  | Duration | Intercept | 0.067 | -0.187 | 0.321 |
|  |  | Weeks | 0.057 | -0.022 | 0.135 |
| Plasma Glucose | Species | Mouse | 0.013 | -0.046 | 0.071 |
|  |  | Rat – Mouse | -0.018 | -0.101 | 0.065 |
|  | Time Fasted | Fasted | 0.009 | -0.037 | 0.056 |
|  |  | Fed – Fasted | -0.025 | -0.124 | 0.074 |
|  | Duration | Intercept | 0.000 | -0.087 | 0.086 |
|  |  | Weeks | 0.002 | -0.038 | 0.042 |
| Plasma Insulin | Species | Mouse | 0.047 | -0.071 | 0.164 |
|  |  | Rat – Mouse | -0.033 | -0.234 | 0.168 |
|  | Time Fasted | Fasted | 0.002 | -0.115 | 0.119 |
|  |  | Fed – Fasted | 0.106 | -0.119 | 0.331 |
|  | Duration | Intercept | -0.046 | -0.239 | 0.147 |
|  |  | Weeks | 0.034 | -0.024 | 0.093 |
| HOMA | Species | Mouse | 0.028 | -0.110 | 0.166 |
|  |  | Rat – Mouse | -0.054 | -0.401 | 0.292 |
|  | Time Fasted | Fasted | 0.061 | -0.091 | 0.214 |
|  |  | Fed – Fasted | -0.136 | -0.410 | 0.138 |
|  | Duration | Intercept | -0.064 | -0.629 | 0.500 |
|  |  | Weeks | 0.026 | -0.131 | 0.183 |
| Mass | **Species** | **Mouse** | **-0.060** | **-0.132** | **0.012** |
|  |  | **Rat – Mouse** | **0.088** | **0.007** | **0.169** |
|  | Duration | Intercept | -0.024 | -0.075 | 0.026 |
|  |  | Weeks | 0.026 | -0.003 | 0.055 |
| Percent Fat Mass | Species | Mouse | 0.042 | -0.100 | 0.184 |
|  |  | Rat – Mouse | -0.113 | -0.418 | 0.193 |
|  | Duration | Intercept | -0.233 | -0.551 | 0.085 |
|  |  | Weeks | 0.067 | -0.014 | 0.148 |
| Food Intake | Species | Mouse | -0.077 | -0.154 | 0.001 |
|  |  | Rat – Mouse | 0.013 | -0.077 | 0.102 |
|  | Duration | Intercept | -0.083 | -0.137 | -0.028 |
|  |  | Weeks | 0.013 | -0.018 | 0.043 |
| Energy Intake | Species | Mouse | -0.065 | -0.135 | 0.006 |
|  |  | Rat – Mouse | 0.009 | -0.073 | 0.090 |
|  | Duration | Intercept | -0.082 | -0.129 | -0.034 |
|  |  | Weeks | 0.020 | -0.008 | 0.047 |

**Table S4**

Results of publication bias tests for each trait. ER is the coefficient from Egger’s regression, alongside the upper and lower 95% confidence limits (ER LCL and ER UCL). Where the ER was considered significant (i.e., LCL to UCL excludes zero) trim and fill analysis was applied. The number of missing studies estimated by the trim and fill analysis is shown, and where missing studies were inferred an adjustment value for the meta-analysis of the trait is given.

| Trait | ER | ER LCL | ER UCL | # Missing | Adjustment | Adj. LCL | Adj. UCL |
| --- | --- | --- | --- | --- | --- | --- | --- |
| Plasma BCAA | -0.639 | -1.029 | -0.249 | 120 | 0.043 | 0.008 | 0.078 |
| Plasma Isoleucine | -0.052 | -0.624 | 0.520 | NA | NA | NA | NA |
| Plasma Leucine | 0.475 | 0.139 | 0.811 | 8 | -0.084 | -0.166 | -0.002 |
| Plasma Valine | -0.378 | -0.729 | -0.026 | 133 | 0.151 | 0.114 | 0.188 |
| Glucose AUC | 0.434 | -0.297 | 1.164 | NA | NA | NA | NA |
| Plasma Glucose | -0.290 | -0.888 | 0.309 | NA | NA | NA | NA |
| Plasma Insulin | 1.171 | 0.485 | 1.858 | 73 | -0.101 | -0.156 | -0.047 |
| HOMA | 2.097 | 1.352 | 2.842 | 57 | -0.030 | -0.099 | 0.040 |
| Mass | 0.085 | -0.443 | 0.613 | NA | NA | NA | NA |
| Percent Fat Mass | 1.133 | 0.331 | 1.934 | 0 | NA | NA | NA |
| Food Intake | 0.003 | -0.255 | 0.261 | NA | NA | NA | NA |
| Energy Intake | -1.683 | -2.945 | -0.421 | 0 | NA | NA | NA |

**Table S5**

Model coefficients for AIC favoured meta-regression for each trait. BS indicates a basis spline, diff indicates the difference between experimental and control diets, LCL and UCL is the lower and upper 95% confidence limit.

| Trait | Coefficient | Estimate | LCL | UCL |
| --- | --- | --- | --- | --- |
| Plasma BCAAs | Intercept | 0.585 | 0.287 | 0.883 |
|  | BS(Diet_BCAA_kJ_g)1 | -0.530 | -0.896 | -0.164 |
|  | BS(Diet_BCAA_kJ_g)2 | -0.855 | -1.314 | -0.397 |
|  | BS(Diet_BCAA_kJ_g)3 | -0.207 | -0.696 | 0.281 |
| Plasma Isoleucine | Intercept | 0.137 | -0.004 | 0.278 |
|  | Diet_Iso_kJ_g | -1.246 | -1.740 | -0.753 |
|  | diff_Iso | 0.991 | 0.614 | 1.367 |
|  | Diet_Iso_kJ_g:diff_Iso | 0.623 | -1.015 | 2.262 |
| Plasma Leucine | Intercept | 0.241 | 0.090 | 0.393 |
|  | Diet_Leu_kJ_g | -0.475 | -0.680 | -0.270 |
|  | diff_Leu | 0.515 | 0.333 | 0.697 |
|  | Diet_Leu_kJ_g:diff_Leu | 0.166 | -0.160 | 0.492 |
| Plasma Valine | Intercept | 0.358 | 0.204 | 0.512 |
|  | Diet_Val_kJ_g | -1.851 | -2.386 | -1.316 |
|  | diff_Val | 0.549 | 0.158 | 0.940 |
|  | Diet_Val_kJ_g:diff_Val | 0.486 | -1.280 | 2.252 |
| Glucose AUC | Intercept | -0.053 | -0.155 | 0.048 |
|  | diff_nonBCAA | 0.129 | 0.053 | 0.204 |
|  | diff_BCAA | 0.351 | 0.048 | 0.655 |
|  | diff_nonBCAA:diff_BCAA | -0.072 | -0.112 | -0.033 |
| Plasma Insulin | Intercept | 0.424 | 0.288 | 0.561 |
|  | BS(Diet_PC_kJ_g)1 | -2.084 | -2.660 | -1.508 |
|  | BS(Diet_PC_kJ_g)2 | 1.421 | 0.765 | 2.078 |
|  | BS(Diet_PC_kJ_g)3 | 0.513 | 0.152 | 0.874 |
| HOMA | Intercept | 1.006 | 0.653 | 1.358 |
|  | BS(Diet_P_kJ_g)1 | -2.123 | -2.841 | -1.406 |
|  | BS(Diet_P_kJ_g)2 | -1.521 | -2.390 | -0.652 |
|  | BS(Diet_P_kJ_g)3 | -0.456 | -1.024 | 0.113 |
| Mass | Intercept | 0.223 | 0.170 | 0.276 |
|  | BS(Diet_BCAA_kJ_g)1 | -0.726 | -0.876 | -0.575 |
|  | BS(Diet_BCAA_kJ_g)2 | 0.107 | -0.040 | 0.254 |
|  | BS(Diet_BCAA_kJ_g)3 | -0.309 | -0.490 | -0.127 |
| Percent Fat Mass | Intercept | -0.231 | -0.374 | -0.087 |
|  | Diet_PC_kJ_g | 0.279 | 0.199 | 0.360 |
|  | diff_BCAA | 0.249 | 0.153 | 0.344 |
|  | Diet_PC_kJ_g:diff_BCAA | 0.016 | -0.106 | 0.139 |
| Food Intake | Intercept | 1.368 | 1.107 | 1.628 |
|  | BS(diff_BCAA.Non_BCAA_kJ_g)1 | -2.662 | -3.188 | -2.136 |
|  | BS(diff_BCAA.Non_BCAA_kJ_g)2 | -0.393 | -0.683 | -0.103 |
|  | BS(diff_BCAA.Non_BCAA_kJ_g)3 | -2.341 | -2.685 | -1.996 |
| Energy Intake | Intercept | 0.951 | 0.685 | 1.217 |
|  | BS(diff_BCAA.Non_BCAA_kJ_g)1 | -1.883 | -2.417 | -1.350 |
|  | BS(diff_BCAA.Non_BCAA_kJ_g)2 | -0.163 | -0.470 | 0.144 |
|  | BS(diff_BCAA.Non_BCAA_kJ_g)3 | -1.889 | -2.247 | -1.531 |

**Table S6.**

Details of articles analysed as indicated in Table S1.

| **Article** | **Reference** |
| --- | --- |
| 1 | Adechian S, Giardina S, Remond D, Papet I, Buonocore D, Gaudichon C, Dardevet D, Marzatico F, Mosoni L: **Excessive Energy Intake Does Not Modify Fed-state Tissue Protein Synthesis Rates in Adult Rats**. *Obesity* 2009, **17**(7):1348-1355. |
| 2 | Amaral AG, Rafacho A, de Oliveira CAM, Batista TM, Ribeiro RA, Latorraca MQ, Boschero AC, Carneiro EM: **Leucine Supplementation Augments Insulin Secretion in Pancreatic Islets of Malnourished Mice**. *Pancreas* 2010, **39**(6):847-855. |
| 3 | Anderson PA, Alster JM, Clinton SK, Imrey PB, Mangian HJ, Truex CR, Visek WJ: **Plasma amino acids and excretion of protein end products by mice fed 10 or 40% soybean protein diets with or without dietary benzo[a]pyrene or 1,2-dimethylhydrazine**. *J Nutr* 1985, **115**(11):1515-1527. |
| 4 | Anderson SA, Tews JK, Harper AE: **Dietary branched-chain amino-acids and protein selection by rats**. *J Nutr* 1990, **120**(1):52-63. |
| 5 | Anderson HL, Benevenga NJ, Harper AE: **Associations among food and protein intake, serine dehydratase, and plasma amino acids**. *Am J Physiol* 1968, **214**(5):1008-1013. |
| 6 | Ascencio C, Torres N, Sandoval RL, Cruz C, Pedraza-Chaverri J, Tovar AR: **Reduced kidney branched chain aminotransferase expression in puromycin aminonucleoside-induced nephrotic syndrome**. *Life Sci* 1997, **61**(24):2407-2415. |
| 7 | Bajotto G, Sato Y, Kitaura Y, Shimomura Y: **Effect of branched-chain amino acid supplementation during unloading on regulatory components of protein synthesis in atrophied soleus muscles**. *Eur J Appl Physiol* 2011, **111**(8):1815-1828. |
| 8 | Bassil MS, Hwalla N, Obeid OA: **Meal pattern of male rats maintained on histidine-, leucine-, or tyrosine-supplemented diet**. *Obesity* 2007, **15**(3):616-623. |
| 9 | Batistela E, Pereira MP, Siqueira JT, Paula-Gomes S, Zanon NM, Oliveira EB, Navegantes LCC, Kettelhut IC, Andrade CMB, Kawashita NH *et al*: **Decreased rate of protein synthesis, caspase-3 activity, and ubiquitin-proteasome proteolysis in soleus muscles from growing rats fed a low-protein, high-carbohydrate diet**. *Can J Physiol Pharmacol* 2014, **92**(6):445-454. |
| 10 | Baum JI, Washington TA, Shouse SA, Bottje W, Dridi S, Davis G, Smith D: **Leucine supplementation at the onset of high-fat feeding does not prevent weight gain or improve glycemic regulation in male Sprague-Dawley rats**. *J Physiol Biochem* 2016, **72**(4):781-789. |
| 11 | Benton DA, Harper AE, Spivey HE, Elvehjem CA: **Leucine, isoleucine and valine relationships in the rat**. *Arch Biochem Biophys* 1956, **60**(1):147-155. |
| 12 | Bhargava HN, Bansinath M, Das S, Matwyshyn GA: **Multiple opiate receptors and pharmacological response to morphine in rats maintained on diets differing in protein concentration**. *Gen Pharmacol* 1987, **18**(5):505-512. |
| 13 | Block KP, Soemitro S, Heywood BW, Harper AE: **Activation of liver branched-chain alpha-keto acid dehydrogenase in rats by excesses of dietary amino acids**. *J Nutr* 1985, **115**(12):1550-1561. |
| 14 | Block KP, Aftring RP, Mehard WB, Buse MG: **Modulation of rat skeletal muscle branched-chain alpha-keto acid dehydrogenase in vivo. Effects of dietary protein and meal consumption**. *J Clin Invest* 1987, **79**(5):1349-1358. |
| 15 | Block KP, Aftring RP, Buse MG: **Regulation of rat liver branched-chain alpha-keto acid dehydrogenase activity by meal frequency and dietary protein**. *J Nutr* 1990, **120**(7):793-799. |
| 16 | Block KP, Harper AE: **High-levels of dietary amino and branched-chain alpha-keto acids alter plasma and brain amino-acid-concentrations in rats**. *J Nutr* 1991, **121**(5):663-671. |
| 17 | Bong HY, Kim JY, Jeong HI, Moon MS, Kim J, Kwon O: **Effects of corn gluten hydrolyzates, branched chain amino acids, and leucine on body weight reduction in obese rats induced by a high fat diet**. *Nutr Res Pract* 2010, **4**(2):106-113. |
| 18 | Bruckbauer A, Zemel MB, Thorpe T, Akula MR, Stuckey AC, Osborne D, Martin EB, Kennel S, Wall JS: **Synergistic effects of leucine and resveratrol on insulin sensitivity and fat metabolism in adipocytes and mice**. *Nutr Metab* 2012, **9**. |
| 19 | de Campos-Ferraz PL, Ribeiro SML, Luz SD, Lancha AH, Tirapegui J: **Exercise x BCAA supplementation in young trained rats: what are their effects on body growth?** *J Sports Sci Med* 2011, **10**(3):483-490. |
| 20 | Caraballo SCG, Comhair TM, Houten SM, Dejong CHC, Lamers WH, Koehler SE: **High-protein diets prevent steatosis and induce hepatic accumulation of monomethyl branched-chain fatty acids**. *J Nutr Biochem* 2014, **25**(12):1263-1274. |
| 21 | Chee KM, Romsos DR, Bergen WG: **Effect of dietary fat on protein intake regulation in young obese and lean mice**. *J Nutr* 1981, **111**(4):668-677. |
| 22 | Chevalier P, Aschkenasy A: **Hematological and immunological effects of excess dietary leucine in the young rat**. *Am J Clin Nutr* 1977, **30**(10):1645-1654. |
| 23 | Colombo JP, Cervantes H, Kokorovic M, Pfister U, Perritaz R: **Effect of different protein diets on the distribution of amino acids in plasma, liver and brain in the rat**. *Ann Nutr Metab* 1992, **36**(1):23-33. |
| 24 | Cummings NE, Williams EM, Kasza I, Konon EN, Schaid MD, Schmidt BA, Poudel C, Sherman DS, Yu D, Arriola Apelo SI *et al*: **Restoration of metabolic health by decreased consumption of branched-chain amino acids**. *J Physiol* 2018, **596**(4):623-645. |
| 25 | da Silva PM, Batista TM, Ribeiro RA, Zoppi CC, Boschero AC, Carneiro EM: **Decreased insulin secretion in islets from protein malnourished rats is associated with impaired glutamate dehydrogenase function: effect of leucine supplementation**. *Metab: Clin Exp* 2012, **61**(5):721-732. |
| 26 | dos Santos GB, de Oliveira AG, Marcondes M, Areas MA: **Long-term Leucine Supplementation Improves Metabolic But Not Molecular Responses in the Skeletal Muscle of Trained Rats Submitted to Exhaustive Exercise**. *J Am Coll Nutr* 2017, **36**(2):81-87. |
| 27 | Etxeberria U, De La Garza AL, Alfredo Martinez J, Milagro FI: **Biocompounds attenuating the development of obesity and insulin resistance produced by a high-fat sucrose diet**. *Nat Prod Commun* 2015, **10**(8):1417-1420. |
| 28 | Filiputti E, Rafacho A, Araujo EP, Silveira LR, Trevisan A, Batista TM, Curi R, Velloso LA, Quesada I, Boschero AC *et al*: **Augmentation of insulin secretion by leucine supplementation in malnourished rats: possible involvement of the phosphatidylinositol 3-phosphate kinase/mammalian target protein of rapamycin pathway**. *Metab: Clin Exp* 2010, **59**(5):635-644. |
| 29 | Fontana L, Cummings NE, Apelo SIA, Neuman JC, Kasza I, Schmidt BA, Cava E, Spelta F, Tosti V, Syed FA *et al*: **Decreased Consumption of Branched-Chain Amino Acids Improves Metabolic Health**. *Cell Rep* 2016, **16**(2):520-530. |
| 30 | French WW, Dridi S, Shouse SA, Wu H, Hawley A, Lee SO, Gu X, Baum JI: **A high-protein diet reduces weight gain, decreases food intake, decreases liver fat deposition, and improves markers of muscle metabolism in obese Zucker rats**. *Nutrients* 2017, **9**(6). |
| 31 | Freudenberg A, Petzke KJ, Klaus S: **Comparison of high-protein diets and leucine supplementation in the prevention of metabolic syndrome and related disorders in mice**. *J Nutr Biochem* 2012, **23**(11):1524-1530. |
| 32 | Freudenberg A, Petzke KJ, Klaus S: **Dietary L-leucine and L-alanine supplementation have similar acute effects in the prevention of high-fat diet-induced obesity**. *Amino Acids* 2013, **44**(2):519-528. |
| 33 | Fu L, Bruckbauer A, Li F, Cao Q, Cui X, Wu R, Shi H, Zemel MB, Xue B: **Leucine amplifies the effects of metformin on insulin sensitivity and glycemic control in diet-induced obese mice**. *Metab: Clin Exp* 2015, **64**(7):845-856. |
| 34 | Fu L, Li F, Bruckbauer A, Cao Q, Cui X, Wu R, Shi H, Xue B, Zemel MB: **Interaction between leucine and phosphodiesterase 5 inhibition in modulating insulin sensitivity and lipid metabolism**. *Diabetes Metab Syndr Obes* 2015, **8**:227-239. |
| 35 | Harper AE, Benjamin E: **Relationship between intake and rate of oxidation of leucine and alpha-ketoisocaproate in vivo in the rat**. *J Nutr* 1984, **114**(2):431-440. |
| 36 | Hashimoto N, Hara H: **Dietary branched-chain amino acids suppress the expression of pancreatic amylase mRNA in rats**. *Biosci Biotechnol Biochem* 2004, **68**(5):1067-1072. |
| 37 | Imamura W, Yoshimura R, Takai M, Yamamura J, Kanamoto R, Kato H: **Adverse Effects of Excessive Leucine Intake Depend on Dietary Protein Intake: A Transcriptomic Analysis to Identify Useful Biomarkers**. *J Nutr Sci Vitaminol* 2013, **59**(1):45-55. |
| 38 | Jacoby SL, Fisher H: **Effect of different carbohydrates on growth rate and branched-chain amino acid requirements in the young adult rat**. *Nutr Rep Int* 1979, **20**(2):145-157. |
| 39 | Kaimoto T, Shibuya M, Nishikawa K, Maeda H: **High incidence of lipid deposition in the liver of rats fed a diet supplemented with branched-chain amino acids under vitamin B6 deficiency**. *J Nutr Sci Vitaminol* 2013, **59**(1):73-78. |
| 40 | Katayama T, Hayashi J, Kishida M, Kato N: **Effects of dietary excess amino acids on the concentrations of cholesterol, alpha-tocopherol, ascorbic acid, and copper in serum and tissues of rats**. *J Nutr Sci Vitaminol* 1990, **36**(5):485-495. |
| 41 | Kim Y, Men SS, Liang C, Receno CN, Brutsaert TD, Korol DL, Heffernan KS, DeRuisseau KC: **Effects of long-term exposures to low iron and branched-chain amino acid containing diets on aging skeletal muscle of Fisher 344 × Brown Norway rats**. *Appl Physiol Nutr Metab* 2018, **43**(2):165-173. |
| 42 | Kobayashi T, Otsuka SI: **Effect of Dietary Excess and Deficiency of Individual Amino Acids on Performance and Protein- and Energy-retention in Growing Rats**. *Agric Biol Chem* 1979, **43**(3):511-516. |
| 43 | Konishi Y, Fuwa H: **Effects of Leucine/Isoleucine Ratio in Amino Acid Mixture-Diets Simulating Normal and High-Lysine Maize Proteins on Growth, Nitrogen Balance, and Tryptophan-Niacin Metabolism in Rats**. *J Nutr Sci Vitaminol* 1982, **28**(6):609-620. |
| 44 | Krauss RM, Mayer J: **Influence of protein and amino acids on food intake in the rat**. *Am J Physiol* 1965, **209**(3):479-483. |
| 45 | Laeger T, Reed SD, Henagan TM, Fernandez DH, Taghavi M, Addington A, Munzberg H, Martin RJ, Hutson SM, Morrison CD: **Leucine acts in the brain to suppress food intake but does not function as a physiological signal of low dietary protein**. *Am J Physiol* 2014, **307**(3):R310-R320. |
| 46 | Li X, Wang X, Liu R, Ma Y, Guo H, Hao L, Yao P, Liu L, Sun X, He K *et al*: **Chronic leucine supplementation increases body weight and insulin sensitivity in rats on high-fat diet likely by promoting insulin signaling in insulin-target tissues**. *Mol Nutr Food Res* 2013, **57**(6):1067-1079. |
| 47 | Liu KA, Lashinger LM, Rasmussen AJ, Hursting SD: **Leucine supplementation differentially enhances pancreatic cancer growth in lean and overweight mice**. *Cancer and Metabolism* 2014, **2 (1) (no pagination)**(6). |
| 48 | Liu R, Li H, Fan W, Jin Q, Chao T, Wu Y, Huang J, Hao L, Yang X: **Leucine supplementation differently modulates branched-chain amino acid catabolism, mitochondrial function and metabolic profiles at the different stage of insulin resistance in rats on high-fat diet**. *Nutrients* 2017, **9**(6). |
| 49 | Lollo PCB, Silva LBC, Batista TM, Morato PN, Moura CS, Cruz AG, Faria JAF, Carneiro EM, Amaya-Farfan J: **Effects of whey protein and casein plus leucine on diaphragm the mTOR pathway of sedentary, trained rats**. *Food Res Intl* 2012, **49**(1):416-424. |
| 50 | Lollo PCB, Batista TM, Moura CS, Morato PN, Cruz AG, Faria JAF, Carneiro EM, Amaya-Farfan J: **L-Leucine supplemented whey protein. Dose-response effect on heart mTOR activation of sedentary and trained rats**. *Food Res Intl* 2013, **53**(1):543-550. |
| 51 | Mandal TK, Parvin N, Mondal S, Saxena V, Saxena AK, Sarkar S, Saha M: **Relation of soya bean meal level to the concentration of plasma free amino acids and body growth in white rats**. *J Anim Physiol Anim Nutr* 2012, **96**(2):191-197. |
| 52 | Margolis LM, Rivas DA, Berrone M, Ezzyat Y, Young AJ, McClung JP, Fielding RA, Pasiakos SM: **Prolonged Calorie Restriction Downregulates Skeletal Muscle mTORC1 Signaling Independent of Dietary Protein Intake and Associated microRNA Expression**. *Front Physiol* 2016, **7**. |
| 53 | Matsuzaki K, Kato H, Sakai R, Toue S, Amao M, Kimura T: **Transcriptomics and metabolomics of dietary leucine excess**. *J Nutr* 2005, **135**(6):1571S-1575S. |
| 54 | May RC, Piepenbrock N, Kelly RA, Mitch WE: **Leucine-induced amino acid antagonism in rats: Muscle valine metabolism and growth impairment**. *J Nutr* 1991, **121**(3):293-301. |
| 55 | Mercer LP, Yi T, Dodds SJ: **Determination of nutritional-requirements in rats - variation with time of weight-gain responses to indispensable amino-acids**. *J Nutr* 1993, **123**(5):964-971. |
| 56 | Mittal PC: **Response of rats to variations in dietary protein content**. *Nutr Rep Int* 1985, **31**(3):521-533. |
| 57 | Morens C, Keijzer M, De Vries K, Scheurink A, Van Dijk G: **Effects of high-fat diets with different carbohydrate-to-protein ratios on energy homeostasis in rats with impaired brain melanocortin receptor activity**. *Am J Physiol* 2005, **289**(1 58-1):R156-R163. |
| 58 | Morrison CD, Xi A, White CL, Ye J, Martin RJ: **Amino acids inhibit Agrp gene expression via an mTOR-dependent mechanism**. *Am J Physiol* 2007, **293**(1):E165-E171. |
| 59 | Mu WC, Vanhoosier E, Elks CM, Grant RW: **Long-term effects of dietary protein and branched-chain amino acids on metabolism and inflammation in mice**. *Nutrients* 2018, **10**(7). |
| 60 | Muramatsu K, Odagiri H, Morishita S, Takeuchi H: **Effect of excess levels of individual amino acids on growth of rats fed casein diets**. *J Nutr* 1971, **101**(9):1117-1125. |
| 61 | Nakagawa I, Sasaki A: **Effect of an excess intake of leucine, with and without additions of vitamin B6 and/or niacin, on tryptophan and niacin metabolism in rats**. *J Nutr Sci Vitaminol* 1977, **23**(6):535-548. |
| 62 | Nakazato K, Hirose T, Song HS: **Increased myostatin synthesis in rat gastrocnemius muscles under high-protein diet**. *Int J Sport Nutr Exerc Metab* 2006, **16**(2):153-165. |
| 63 | Nassl AM, Rubio-Aliaga I, Sailer M, Daniel H: **The Intestinal Peptide Transporter PEPT1 Is Involved in Food Intake Regulation in Mice Fed a High-Protein Diet**. *PLoS ONE* 2011, **6**(10). |
| 64 | Noatsch A, Petzke KJ, Millrose MK, Klaus S: **Body weight and energy homeostasis was not affected in C57BL/6 mice fed high whey protein or leucine-supplemented low-fat diets**. *Eur J Nutr* 2011, **50**(6):479-488. |
| 65 | Noguchi Y, Nishikata N, Shikata N, Kimura Y, Aleman JO, Young JD, Koyama N, Kelleher JK, Takahashi M, Stephanopoulos G: **Ketogenic essential amino acids modulate lipid synthetic pathways and prevent hepatic steatosis in mice**. *PLoS ONE* 2010, **5**(8). |
| 66 | Norton LE, Wilson GJ, Moulton CJ, Layman DK: **Meal Distribution of Dietary Protein and Leucine Influences Long-Term Muscle Mass and Body Composition in Adult Rats**. *J Nutr* 2017, **147**(2):195-201. |
| 67 | Ohguri S: **Effects of excess leucine on growth and tryptophan and niacin metabolism in rats**. *J Nutr Sci Vitaminol* 1980, **26**(2):141-160. |
| 68 | Oku H, Urahashi A, Chinen I: **Correlation between serum concentrations of the branched-chain amino-acid and alpha-keto-acid and the distribution of the branched-chain fatty-acids on rat skin surface**. *J Nutr Sci Vitaminol* 1991, **37**(3):297-311. |
| 69 | Owens FN, Shin S, Pettigrew JE, Oltjen JW: **Apportioning leucine requirements for maintenance versus growth for rats**. *Nutr Res* 1994, **14**(1):73-82. |
| 70 | Peters JC, Harper AE: **Adaptation of rats to diets containing different levels of protein: Effects on food intake, plasma and brain amino acid concentrations and brain neurotransmitter metabolism**. *J Nutr* 1985, **115**(3):382-398. |
| 71 | Pick RT, Meade RJ: **Amino acid supplementation of opaque-2 corn diets for growing rats**. *J Nutr* 1971, **101**(9):1241-1248. |
| 72 | Ramadan NG, Mattar MA, Obeid OA: **Meal pattern of male rats maintained on individual branched chain amino acid supplemented diet**. *Curr Top Nutraceutical Res* 2009, **7**(3-4):149-156. |
| 73 | Rogers QR, Tannous RI, Harper AE: **Effects of excess leucine on growth and food selection**. *J Nutr* 1967, **91**(4):561-572. |
| 74 | Sanahuja JC, Rio ME, Lede MN: **Decrease in Appetite and Biochemical Changes in Amino Acid Imbalance in the Rat**. *J Nutr* 1965, **86**(4):424-432. |
| 75 | Scharrer E, Baile CA, Mayer J: **Effect of amino acids and protein on foot intake of hyperphagic and recovered aphagic rats**. *Am J Physiol* 1970, **218**(2):400-404. |
| 76 | Shimomura Y, Murakami T, Nakai N, Nagasaki M, Obayashi M, Li Z, Xu M, Sato Y, Kato T, Shimomura N *et al*: **Suppression of glycogen consumption during acute exercise by dietary branched-chain amino acids in rats**. *J Nutr Sci Vitaminol* 2000, **46**(2):71-77. |
| 77 | Shin IS, Owens FN, Pettigrew JE, Oltjen JW: **Apportioning valine requirements for maintenance versus growth for rats**. *Nutr Res* 1994, **14**(1):117-126. |
| 78 | Soemitro S, Block KP, Crowell PL, Harper AE: **Activities of branched-chain amino acid-degrading enzymes in liver from rats fed different dietary levels of protein**. *J Nutr* 1989, **119**(8):1203-1212. |
| 79 | Solon-Biet SM, McMahon AC, Ballard JW, Ruohonen K, Wu LE, Cogger VC, Warren A, Huang X, Pichaud N, Melvin RG *et al*: **The ratio of macronutrients, not caloric intake, dictates cardiometabolic health, aging, and longevity in ad libitum-fed mice**. *Cell Metab* 2014, **19**(3):418-430. |
| 80 | Solon-Biet SM, Cogger VC, Pulpitel T, Wahl D, Clark X, Bagley EE, Gregoriou GC, Senior AM, Wang Q-P, Brandon AE *et al*: **Branched-chain amino acids impact health and lifespan indirectly via amino acid balance and appetite control**. *Nat Metab* 2019, **1**(5):532-545. |
| 81 | Spolter PD, Harper AE: **Leucine-iso leucine antagonism in the rat**. *Am J Physiol* 1961, **200**(3):513-518. |
| 82 | Spolter PD, Harper AE: **Adaptation of rats to diets containing ethionine or excess leucine**. *J Nutr* 1963, **80**(4):386-390. |
| 83 | Tanaka H, Yamaguchi M, Kametaka M: **Metabolism of leucine and alanine in growing rats fed the diets with various protein to energy ratios**. *Agric Biol Chem* 1975, **39**(2):507-514. |
| 84 | Tanaka H, Fukushima T, Nakatomi Y, Ogura M: **Metabolism of Valine and Isoleucine in Growing Rats at Various Dietary Protein Levels**. *Agric Biol Chem* 1988, **52**(3):811-817. |
| 85 | Taniguchi H, Akiyama N, Ishihara K: **Effects of dietary protein contents and habitual endurance exercise on supplemental leucine oxidation in mice**. *Biosci Biotechnol Biochem* 2019, **83**(4):728-737. |
| 86 | Toneto AT, Ferreira Ramos LA, Salomao EM, Tomasin R, Aereas MA, Gomes-Marcondes MC: **Nutritional leucine supplementation attenuates cardiac failure in tumour-bearing cachectic animals**. *J Cachexia Sarcopenia Muscle* 2016, **7**(5):577-586. |
| 87 | Torres-Leal FL, Fonseca-Alaniz MH, Teodoro GF, De Capitani MD, Vianna D, Pantaleao LC, Matos-Neto EM, Rogero MM, Donato J, Tirapegui J: **Leucine supplementation improves adiponectin and total cholesterol concentrations despite the lack of changes in adiposity or glucose homeostasis in rats previously exposed to a high-fat diet**. *Nutr Metab* 2011, **8:62**(62). |
| 88 | Vianna D, Resende GF, Torres-Leal FL, Pantaleao LC, Donato J, Jr., Tirapegui J: **Long-term leucine supplementation reduces fat mass gain without changing body protein status of aging rats**. *Nutrition* 2012, **28**(2):182-189. |
| 89 | Weisdorf SA, Hamel N, Pierpont ME, Bowers LD, Cerra FB: **Increased dietary branched-chain amino acids do not improve growth in developing rats with chronic biliary obstruction**. *J Nutr* 1991, **121**(9):1447-1453. |
| 90 | Yamamoto Y: **Self-selection of Dietary Branched-chain Amino Acids by Rats**. *Agric Biol Chem* 1987, **51**(4):1023-1031. |
| 91 | Yoshimura R, Takai M, Namaki H, Minami K, Imamura W, Kato H, Kamei Y, Kanamoto R: **Down Regulation of Asparagine Synthetase and 3-Phosphoglycerate Dehydrogenase, and the Up-Regulation of Serine Dehydratase in Rat Liver from Intake of Excess Amount of Leucine Are Not Related to Leucine-Caused Amino Acid Imbalance**. *J Nutr Sci Vitaminol* 2015, **61**(6):441-448. |
| 92 | Young VR, Zamora J: **Effects of altering the proportions of essential to nonessential amino acids on growth and plasma amino acid levels in the rat**. *J Nutr* 1968, **96**(1):21-27. |
| 93 | Yuan XW, Han SF, Zhang JW, Xu JY, Qin LQ: **Leucine supplementation improves leptin sensitivity in high-fat diet fed rats**. *Food Nutr Res* 2015, **59**. |
