# Supplementary material for: Meta-analysis links dietary branched-chain amino acids to metabolic health in rodents
Source: BMC Biol. 2022 Jan 14;20:19. doi: 10.1186/s12915-021-01201-2 (PMC8760763; doi:10.1186/s12915-021-01201-2)
Supplement: Supplementary file 2 — Additional File 2. Detailed methods. [file 12915_2021_1201_MOESM2_ESM.docx]

**Detailed methods**

Systematic review protocol for animal intervention studies

Search strategy for Web of Science

TOPIC* (diet*) **AND** TOPIC* (“branched chain amino”) OR TOPIC* (BCAA*) OR TOPIC* (*leucine) OR TOPIC (*valine) **AND** TS=(Murinae) OR TS=(Mouse) OR TS=(Rat) OR TS=(Murine) OR TS=(rodent*) OR TS=(mice) OR TS=(rats) OR TS=(mus)

Search Strategy for Scopus

(( TITLE-ABS-KEY ( "branched chain amino" )  OR  TITLE-ABS-KEY ( bcaa* )  OR  TITLE-ABS-KEY ( *leucine )  OR  TITLE-ABS-KEY ( *valine ) ) )  **AND**  ( TITLE-ABS-KEY ( diet* ) )  **AND** ( ( TITLE-ABS-KEY ( murinae )  OR  TITLE-ABS-KEY ( mouse )  OR  TITLE-ABS-KEY ( rat )  OR  TITLE-ABS-KEY ( murine )  OR  TITLE-ABS-KEY ( rodent* )  OR  TITLE-ABS-KEY ( mus ) ) )

Search strategy for Medline

The exposure*

1. "branched chain amino".tw.

2. Murinae/

3. Mice, Congenic/

4. exp Mice, inbred strains/

5. exp Rats, inbred strains/

6. Rats, Long-evans/

7. Rats, Sprague-Dawley/

8. Rats, Wistar/

9. mouse.tw.

10. mice.tw.

11. murine.tw.

12. rats.tw.

13. rat.tw.

14. murinae.tw.

15. murine.tw.

16. mus.tw.

17. Amino Acids, Branched-Chain/

18. BCAA*.tw.

19. Isoleucine.mp.

20. Alloisoleucine.mp.

21. Leucine.mp.

22. Valine.mp.

23. exp Diet/

24. diet*.tw.

25. 23 or 24

26. rodent*.tw.

27. 2 or 3 or 4 or 5 or 6 or 7 or 8 or 9 or 10 or 11 or 12 or 13 or 14 or 15 or 16 or 26

28. 1 or 17 or 18 or 19 or 20 or 21 or 22

29. 25 and 27 and 28

Search strategy for Embase

1. germfree mouse/

2. mouse model/

3. albino mouse/

4. exp dwarf mouse/

5. exp hybrid mouse strain/

6. exp inbred mouse strain/

7. mongrel mouse/

8. exp outbred mouse strain/

9. pigmented mouse/

10. exp random bred mouse/

11. wild type mouse/

12. murine/

13. exp Apodemus/

14. exp Arvicanthis/

15. Bandicata/

16. exp Mastomys/

17. Praomys/

18. Mus booduga/

19. exp Mus Musculus/

20. Mus Spretus/

21. Mus Terricolor/

22. Rattus rattus/

23. rattus norvegicus/

24. germfree rat/

25. rat model/

26. albino rat/

27. dwarf rat/

28. pigmented rat/

29. exp inbred rat strain/

30. mongrel rat/

31. exp outbred rat strain/

32. hooded rat/

33. exp hybrid rat strain/

34. exp random bred rat/

35. wild type rat/

36. mouse.tw.

37. mice.tw.

38. murine.tw.

39. rats.tw.

40. rat.tw.

41. murinae.tw.

42. rodent*.tw.

43. mus.tw.

44. 1 or 2 or 3 or 4 or 5 or 6 or 7 or 8 or 9 or 10 or 11 or 12 or 13 or 14 or 15 or 16 or 17 or 18 or 19 or 20 or 21 or 22 or 23 or 24 or 25 or 26 or 27 or 28 or 29 or 30 or 31 or 32 or 33 or 34 or 35 or 36 or 37 or 38 or 39 or 40 or 41 or 42 or 43

45. exp branched chain amino acid/

46. BCAA*.tw.

47. Isoleucine.mp.

48. Leucine.mp.

49. Valine.mp.

50. "branched chain amino".tw.

51. Alloisoleucine.mp.

52. 45 or 46 or 47 or 48 or 49 or 50 or 51

53. diet*.tw.

54. exp diet/

55. 53 or 54

56. 44 and 52 and 55

**Nutritional Models and Associated Hypotheses**

The competing MLMRs each represent different hypotheses about how the effects of increasing dietary BCAAs on outcomes are dependent on nutritional context. In all cases the effect size of interest is the log response ratio (lnRR). A positive lnRR indicates that the mean outcome of interest is higher in the experimental diet (i.e. the diet with higher in total energy from BCAAs) than the control diet (i.e. the diet with lower total BCAA content), and a negative value *vice versa*.

In part 1 we explored the effects of total dietary BCAAs on circulating levels of total BCAAs and individual BCAAs; here a positive lnRR indicates that on a diet higher in total BCAAs mean circulating amino-acid levels are higher. Models with the following nutritional moderators were explored to evaluate the stated hypotheses*

1. The amount of the focal BCAA of interest in the effects size that is in the control diet.

- Dietary amino acid content is the primary determinant of circulating levels, but there is a non-linear association (e.g. saturation effect); e.g. if a diet is already high in a given BCAA, further elevation is not expected result in large effects.

1. The amount of protein in the control diet.

- Dietary protein content is the primary determinant of circulating levels, but there is a non-linear association (e.g. saturation effect); e.g. if a diet is already high in protein, further elevation is not expected result in large effects.

1. The difference between the amount of the circulating BCAA in the control and experimental diets.

- Dietary amino acid content is the primary determinant of circulating levels, where larger increases lead to the biggest increases in circulating levels.

1. The difference between the amount of protein in the control and experimental diets.

- Dietary protein is the primary determinant of circulating levels, where larger increases lead to the biggest increases in circulating levels.

1. Interaction between 1 and 3.
2. Interaction between 2 and 4.

In parts 2 and 3 we explored the effects of total dietary BCAAs on metabolic health and body composition; here a positive lnRR indicates that on a diet higher in total BCAAs the mean measure of interest is higher. Models with the following nutritional moderators were explored*

1. The amount of total BCAAs in the control diet.

- Dietary BCAA content is the primary determinant of outcome, but there is a non-linear association (e.g. saturation effect); e.g. if a diet is already high in BCAAs, further elevation is not expected result in large effects.

1. The amount of protein in the control diet.

- Dietary protein content is the primary determinant of outcome, but there is a non-linear association (e.g. saturation effect); e.g. if a diet is already high in protein, further elevation is not expected result in large effects.

1. The ratio of protein to carbohydrate in the control diet.
2. The difference between the amount of BCAAs in the control and experimental diets.
3. The difference between the ratio of BCAAs to non-BCAAs in the control and experimental diets.
4. Interaction between 1 and 4.
5. Interaction between 2 and 4.
6. Interaction between 3 and 4.
7. Interaction between the difference between the amount of non-BCAAs in the control and experimental diets and 4.
8. Interaction between the difference in BCAAs and difference in leucine.
9. Interaction between the difference in BCAAs and difference in isoleucine.
10. Interaction between the difference in BCAAs and difference in valine.

- 10 through 12 assess whether the outcome is predominantly affected by a focal BCAA rather than the whole BCAA complex.

As described in the main text, for all models we implemented a version with a linear effect and a non-linear effect (basis spline with 3 degrees of freedom). To compare support for the different hypotheses we compared models via AIC, and included the meta-analytic model as a null model
